# Tick-transmitted thogotovirus gains high virulence by a single MxA escape mutation in the viral nucleoprotein

**DOI:** 10.1371/journal.ppat.1009038

**Published:** 2020-11-16

**Authors:** Jonas Fuchs, Alexander Oschwald, Laura Graf, Georg Kochs

**Affiliations:** 1 Institute of Virology, Medical Center–University of Freiburg, Freiburg, Germany; 2 Faculty of Medicine, University of Freiburg, Freiburg, Germany; Washington University in Saint Louis, UNITED STATES

## Abstract

Infections with emerging and re-emerging arboviruses are of increasing concern for global health. Tick-transmitted RNA viruses of the genus *Thogotovirus* in the *Orthomyxoviridae* family have considerable zoonotic potential, as indicated by the recent emergence of Bourbon virus in the USA. To successfully infect humans, arboviruses have to escape the restrictive power of the interferon defense system. This is exemplified by the high sensitivity of thogotoviruses to the antiviral action of the interferon-induced myxovirus resistance protein A (MxA) that inhibits the polymerase activity of incoming viral ribonucleoprotein complexes. Acquiring resistance to human MxA would be expected to enhance the zoonotic potential of these pathogens. Therefore, we screened a panel of 10 different thogotovirus isolates obtained from various parts of the world for their sensitivity to MxA. A single isolate from Nigeria, Jos virus, showed resistance to the antiviral action of MxA in cell culture and in MxA-transgenic mice, whereas the prototypic Sicilian isolate SiAr126 was fully MxA-sensitive. Further analysis identified two amino acid substitutions (G327R and R328V) in the viral nucleoprotein as determinants for MxA resistance. Importantly, when introduced into SiAr126, the R328V mutation resulted in complete MxA escape of the recombinant virus, without causing any viral fitness loss. The escape mutation abolished viral nucleoprotein recognition by MxA and allowed unhindered viral growth in MxA-expressing cells and in MxA-transgenic mice. These findings demonstrate that thogotoviruses can overcome the species barrier by escaping MxA restriction and reveal that these tick-transmitted viruses may have a greater zoonotic potential than previously suspected.

## Author summary

Thogotovirus infections are known to cause isolated human fatalities, yet the zoonotic potential of these tick-transmitted pathogens is still largely unexplored. In the present study, we examined if these viruses are able to escape the interferon-induced human MxA, thereby overcoming the human innate antiviral defense. Mx proteins constitute a class of interferon-induced antiviral effector molecules that efficiently block the intracellular replication of many viruses. Here, we studied the MxA sensitivity of various thogotovirus isolates and identified two amino acid residues in the viral nucleoprotein that caused resistance to MxA. One of these exchanges was sufficient to enable an otherwise MxA-sensitive thogotovirus to fully escape MxA restriction without causing any fitness loss. Our study explores the interplay of thogotoviruses with the innate antiviral host defense and sheds light on their zoonotic potential.

## Introduction

The family of *Orthomyxoviridae* consists of seven genera: *Influenzavirus A-D*, *Isavirus*, *Quaranfil virus* and *Thogotovirus* [1,2]. Many members are important animal and human pathogens like, for example, the aerosol-transmitted influenza A viruses (IAV) that are responsible for up to 600,000 deaths in the human population annually [3]. Thogotoviruses are unique within this family because they are transmitted by ticks and circulate in diverse mammalian species including rodents, wild big game, sheep, cattle, and camels [4–8], causing a febrile illness and occasional abortions [9]. They phylogenetically cluster into Thogoto (THOV)-like and Dhori (DHOV)-like representatives of the genus *Thogotovirus* [1] and possess an envelope and a segmented single-stranded RNA genome of negative polarity [10]. Their six segments are encapsidated by the viral nucleoprotein (NP) and form together with the viral polymerase replication-competent viral ribonucleoprotein complexes (vRNPs) [11,12]. After receptor-mediated entry, the incoming vRNPs are released into the cytoplasm and translocate to the nucleus where viral transcription and replication is initiated [13]. The newly formed virions are released by budding from the cell membrane and can be transmitted by tick bites to new hosts [13,14].

In the past, occasional thogotovirus infections and disease in humans were reported. The patients regularly developed a febrile illness accompanied by neurological symptoms [15,16]. These infections might be more frequent than previously thought as a recent study from Spain showed a thogotovirus seroprevalence of around 5% in individuals with a history of tick bites [8]. The severity of such a disease was exemplified in 2015 and 2017, when two patients died in the United States due to an infection with Bourbon virus (BRBV), a recent thogotovirus isolate. The disease progression of both patients was characterized by severe respiratory complications and liver damage [17,18]. Epidemiological studies in the Midwest and Southern parts of the United States revealed that BRBV is prevalent in lone star ticks and in wild and domestic animals [5,19–21].

During the last 60 years, 15 different thogotoviruses have been isolated in various parts of the world. However, it remains unclear whether they are capable to infect humans. Successful infection is determined to a large extent by the ability of the infecting virus to cope with the hosts’ innate immune defenses. The interferon (IFN) system acts as a major first line of defense against zoonotic transmissions by providing potent restriction factors with broad antiviral activity [22]. One of the key antiviral effectors against orthomyxoviruses is the IFN-induced myxovirus resistance protein 1 (MX1, designated MxA in humans). MxA is a large dynamin-like GTPase, that forms higher order oligomers, and targets the vRNPs thereby interfering with early steps of the viral life cycle [23,24]. GTP binding and hydrolysis are required for antiviral activity, whereas GTPase-deficient mutants, such as MxA(T103A), are inactive [25]. Previous studies with the THOV-like prototype Sicilian isolate SiAr126 showed that MxA co-precipitates with the viral NP [26,27] and blocks the nuclear import of incoming vRNPs [28]. Infection experiments with MxA-transgenic mice (h*MX1*-tg) revealed the antiviral potency of human MxA *in vivo* because these animals were fully protected against an otherwise lethal challenge with SiAr126 [29].

THOV-like viruses are extremely sensitive to the antiviral action of MxA [29,30]. To unravel possible escape strategies within the genus *Thogotovirus* we analyzed a panel of 10 different isolates collected from various parts of the world for their MxA sensitivities. Our analyses identified a single THOV-like isolate with reduced MxA sensitivity that allowed us to characterize two critical residues in the viral NP responsible for the MxA sensitivity of THOV-like viruses. This knowledge will be instrumental to forecast the MxA sensitivity of future thogotovirus isolates and to evaluate their possible zoonotic potential.

## Results

### MxA sensitivity of diverse thogotovirus isolates

**Fig 1:**
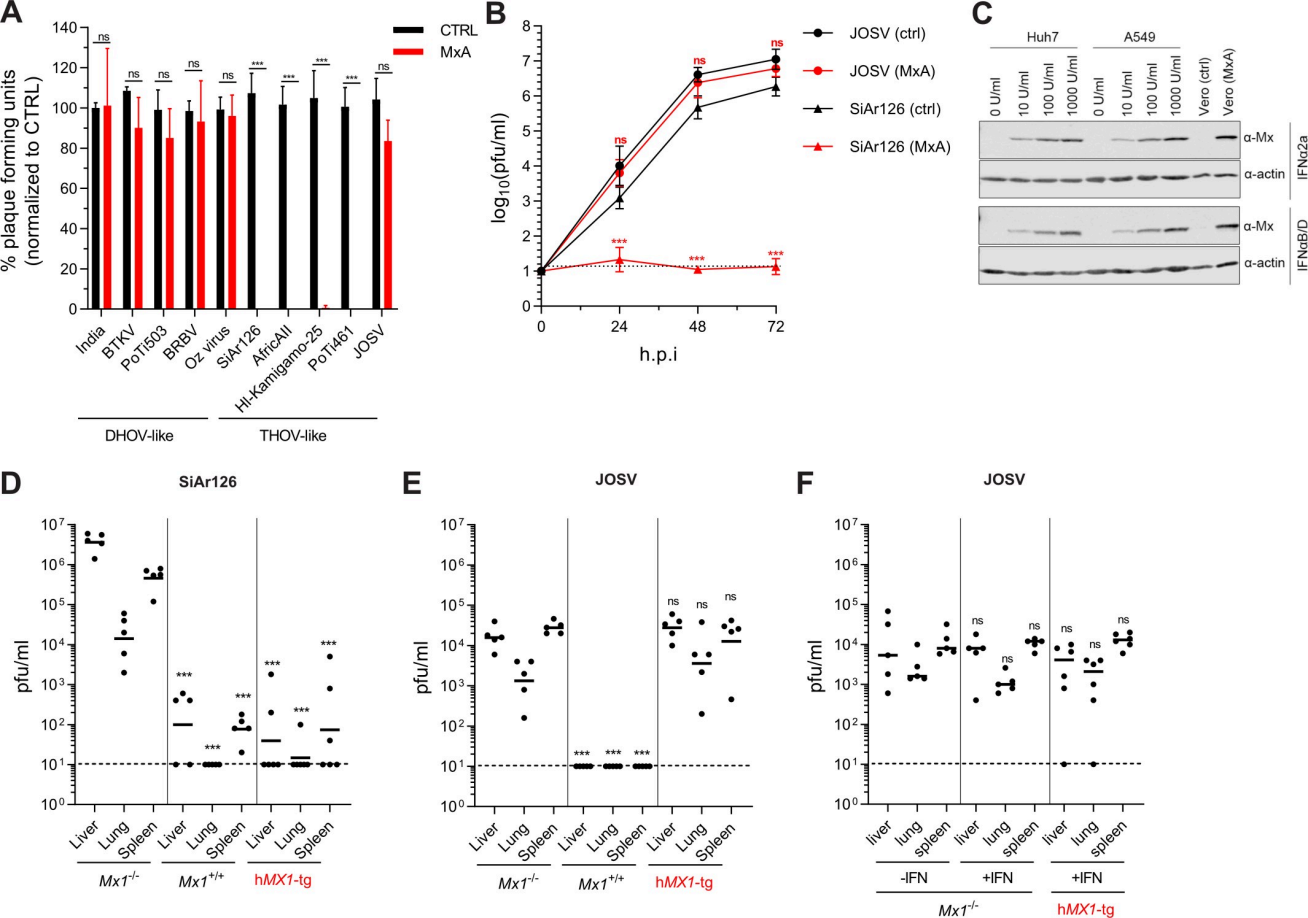
JOSV but not SiAr126 is resistant to the antiviral action of MxA. (A) Vero cells stably expressing MxA (MxA) or control Vero cells (CTRL) were infected with 50 pfu of each virus. A plaque assay was performed to determine the plaque reduction in the presence of MxA. Counted plaques were normalized to the control for each virus (mean ± SD (n = 3), one-way ANOVA, Tukey’s multiple comparison test, ***p<0.001, ns–not significant) (B) Growth kinetics of JOSV and SiAr126 in the presence or absence of MxA. Vero-MxA or control Vero cells were infected with JOSV or SiAr126 (MOI 0.001). At the indicated time points the supernatant was harvested and the viral load determined by plaque assay (mean ± SD (n = 3), two-way ANOVA, Tukey’s multiple comparison test, ***p<0.001, ns–not significant). (C) Western blot analysis of MxA expression in Vero-MxA or control Vero cells in comparison to Huh7 or A549 cells treated with the indicated amounts of human IFNα2a or IFNαB/D for 24 h [42]. (D, E) The *in vivo* protective effect of mouse MX1 and human MxA against SiAr126 and JOSV was analyzed by i.p. infections of MX1-negative C57BL/6 mice (*Mx1*^-/-^), MX1-positive C57BL/6 mice congenic for murine A2G-*Mx1* (*Mx1*^+/+^) or MxA-transgenic mice carrying the human *MX1* locus (h*MX1*-tg^+/+^, red) with 100 pfu of SiAr126 (D) or 1000 pfu of JOSV (E) for 4 days (n = 5). Organs were harvested and the viral load determined by plaque assay. (F) *Mx1*^-/-^ mice or h*MX1*-tg^+/+^ mice were treated i.p. with 20,000 IU IFNαB/D for 16 h prior to infection with 1,000 pfu of JOSV (n = 5) or were left untreated. The viral load in the organs was determined 4 days after infection by plaque assay. (D-F) Shown are the geometric means. Statistical analyses were performed with a one-way ANOVA on log-transformed values (Tukey’s multiple comparison test, ***p<0.001, ns–not significant) and displayed in comparison to the *Mx1*^-/-^ C57BL/6 group (D, E) or untreated animals (F).

We used a collection of 10 different thogotovirus isolates from various parts of the world [4,18,31–38] to investigate their MxA sensitivity. The identity of the virus stocks was confirmed by NGS analysis. In an initial screen, MxA overexpressing and control Vero cells lacking MxA [39] were infected with the isolates using a fixed amount of 50 plaque forming units (pfu) to monitor the virus propagation. All DHOV-like isolates were able to form plaques in the presence of MxA (Fig 1A). In contrast, the THOV-like viruses were potently inhibited by MxA with the striking exception of JOSV (Fig 1A). This surprising MxA insensitivity of JOSV was further investigated by analyzing its growth in control and MxA overexpressing cells. The THOV-like isolate SiAr126 was analyzed in parallel as an MxA-sensitive prototype virus. Both viruses grew to high titers in control cells (Fig 1B). JOSV also replicated to comparable titers in Vero-MxA cells, whereas SiAr126 replication was potently inhibited (Fig 1B). Notably, the level of MxA produced by these stably transduced Vero cells was comparable to endogenous MxA levels of IFNα-treated human Huh7 and A549 cells (Fig 1C).

The MxA insensitivity of JOSV was further analyzed *in vivo*, using experimental infections of C57BL/6 mice that express the entire human *MX1* gene locus as a transgene (h*MX1*-tg) [29]. Conventional C57BL/6 mice carry a defective *Mx1* gene (*Mx1*^-/-^) are susceptible to influenza and other orthomyxoviruses [40], whereas congenic B6.A2G-Mx1 mice (*Mx1*^+/+^) that carry a functional murine *Mx1* are resistant [41] and served as positive controls. Both viruses replicated to high titers in liver, lung and spleen of *Mx1*^-/-^ mice at four days after intraperitoneal (i.p.) infection, but were potently restricted in *Mx1*^+/+^ animals (Fig 1D and 1E). In h*MX1*-tg mice, SiAr126 replication was reduced by up to 100,000 fold, whereas JOSV growth was not affected (Fig 1D and 1E). To exclude the possibility that JOSV failed to induce an IFN response in the h*MX1*-tg animals, we pretreated these animals with IFNαB/D [42] before infection that was shown to induce MxA synthesis [29]. No reductions in viral titers were observed in the pretreated *Mx1*^-/-^ and h*MX1*-tg animals (Fig 1F), confirming the MxA escape of JOSV.

Collectively, these data show that DHOV-like but not THOV-like isolates are resistant to the antiviral action of MxA. One exception is the THOV-like isolate JOSV, which defies MxA both in cell culture and *in vivo*.

### The viral nucleoprotein determines MxA sensitivity

**Fig 2:**
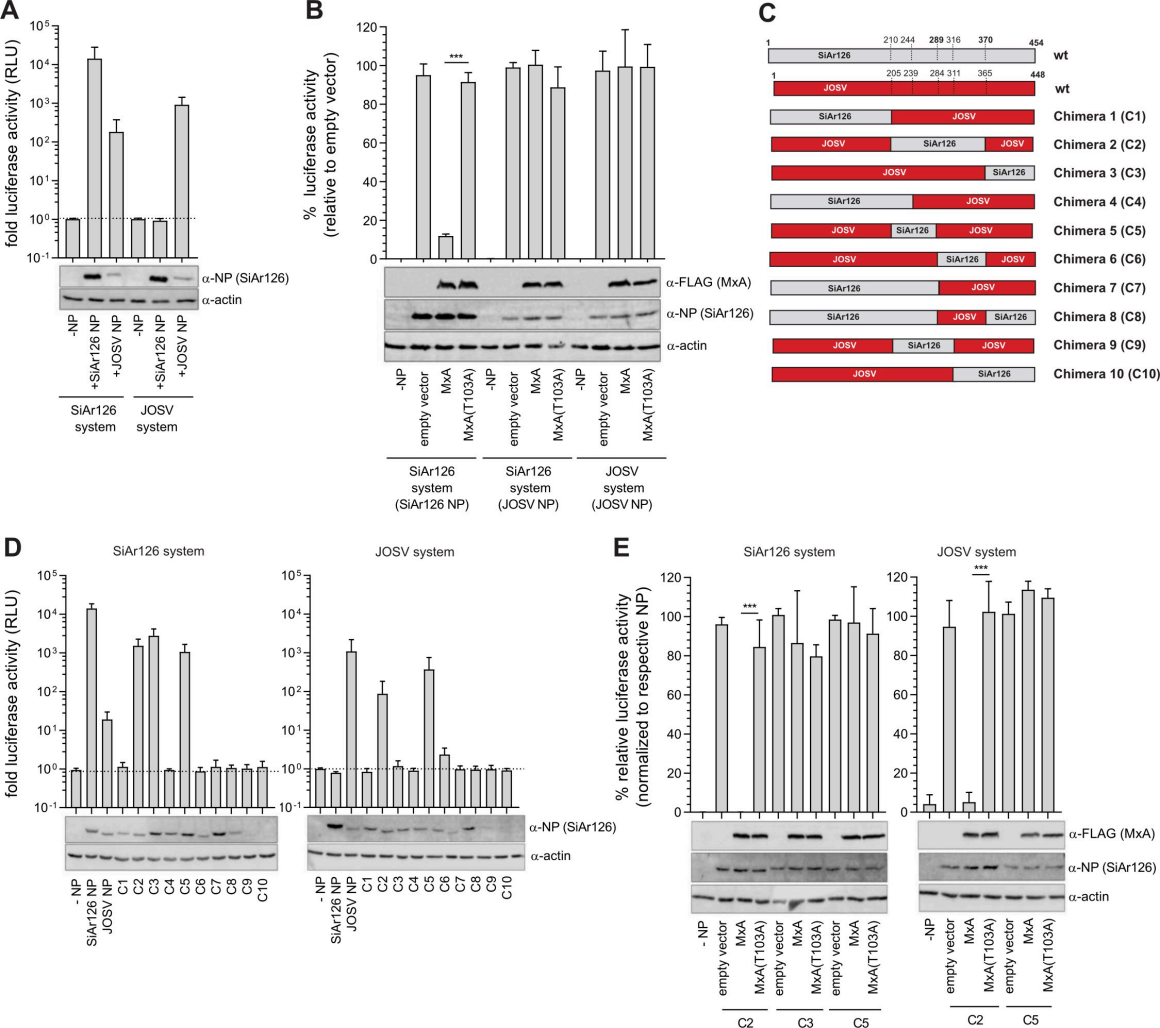
The viral nucleoprotein determines MxA sensitivity. (A-E) 293T cells were co-transfected with the components of the polymerase reconstitution system for either SiAr126 or JOSV, consisting of 10 ng of the expression plasmids encoding the polymerase subunits PB1, PB2, PA, 50 ng for the respective NPs, 50 ng for a viral minigenome coding for firefly luciferase reporter under the control of the viral promotor (pPol-I FF-Luc) and 10 ng for Renilla luciferase under a constitutive promotor (RLuc). 24 h after transfection the cells were lysed and the firefly and Renilla luciferase activities determined. The firefly luciferase was normalized to the Renilla luciferase activity and the expression of NP, actin and MxA was controlled by Western blot. Significance was calculated with a one-way ANOVA (Tukey’s multiple comparison test, ***p<0.001, ns–not significant). (A) Activity of the SiAr126 and JOSV polymerase reconstitution system in the absence of MxA. 293T cells were co-transfected with the components of the SiAr126 or JOSV polymerase reconstitution system. The control without NP (–NP) was set to 1 (mean ± SD, n = 3). (B) Inhibition of the SiAr126 or JOSV system by MxA. The SiAr126 or JOSV systems were co-transfected with 50 ng expression plasmids for human MxA or the antivirally inactive mutant MxA(T103A). The empty vector controls were each set to 100% (mean ± SD, n = 3). (C) Schematic representation of the chimeric viral NPs. Grey represents SiAr126 NP and red JOSV NP. Fusion sites were selected in conserved regions of the proteins and are indicated as dotted lines with the corresponding amino acid positions. (D) Relative luciferase activity of the chimeric NPs in the SiAr126 or JOSV systems in the absence of MxA. The polymerase reconstitution systems were co-transfected with 50 ng of the chimeric NPs. The (–NP) control was set to 1 (mean ± SD, n = 3). (E) MxA sensitivity of the functional chimeric NPs. 293T cells were co-transfected with the components of the SiAr126 or JOSV system including 50 ng of the compatible chimeric NP expression plasmids and 50 ng MxA or MxA(T103A). The empty vector controls for the individual chimeric NPs were set to 100% (mean ± SD, n = 3).

The human MxA protein blocks the polymerase activity of incoming SiAr126 vRNPs by targeting NP [26,27]. Therefore, the antiviral effect of MxA can be studied by analyzing its impact on the activity of a thogotovirus polymerase reconstitution system [43]. In these systems expression of the three viral polymerase subunits and NP leads to the transcription of an artificial viral minigenome that encodes a firefly luciferase reporter flanked by viral non-coding regions [43]. The influence of NP on MxA sensitivity was tested in the established SiAr126 polymerase reconstitution system and in an analogous system for JOSV (Fig 2A). SiAr126 NP and to a lesser degree JOSV NP resulted in firefly luciferase reporter activity in the SiAr126 system. However, the JOSV system was only compatible with JOSV NP but not SiAr126 NP (Fig 2A). Co-expression of MxA reduced the polymerase activity of the authentic SiAr126 system by 90% in comparison to the antivirally inactive mutant MxA(T103A) (Fig 2B). As expected from the cell culture experiments in Fig 1, the polymerase activity of the authentic JOSV system was not affected by MxA (Fig 2B). Moreover, no inhibition by MxA was detectable after changing the NP to the one of JOSV in the SiAr126 system, suggesting that the NP determines MxA sensitivity.

In order to narrow down the region responsible for MxA sensitivity, we constructed plasmids coding for chimeric NPs of JOSV and SiAr126. The fusion sites of the fragments were placed in regions that are highly conserved between both NPs (Fig 2C) (S3 Fig). The resulting chimeras were tested in both polymerase reconstitution systems. Unfortunately, only chimeras C2, C3 and C5 were compatible with the SiAr126 system and C2 and C5 with the JOSV system, as shown by the reporter activity of the respective viral polymerase reconstitution system (Fig 2D). Interestingly, MxA inhibited the polymerase activity if C2, but not if C3 or C5 were used in the SiAr126 system (Fig 2E). Accordingly, the JOSV polymerase was MxA-sensitive if C2 but not if C5 was expressed (Fig 2E). Since the regions of SiAr126 NP overlap in construct C2 and C5, we concluded that the NP determinant responsible for MxA sensitivity of SiAr126 is located between amino acids 289 to 370 (Fig 2C).

**Fig 3:**
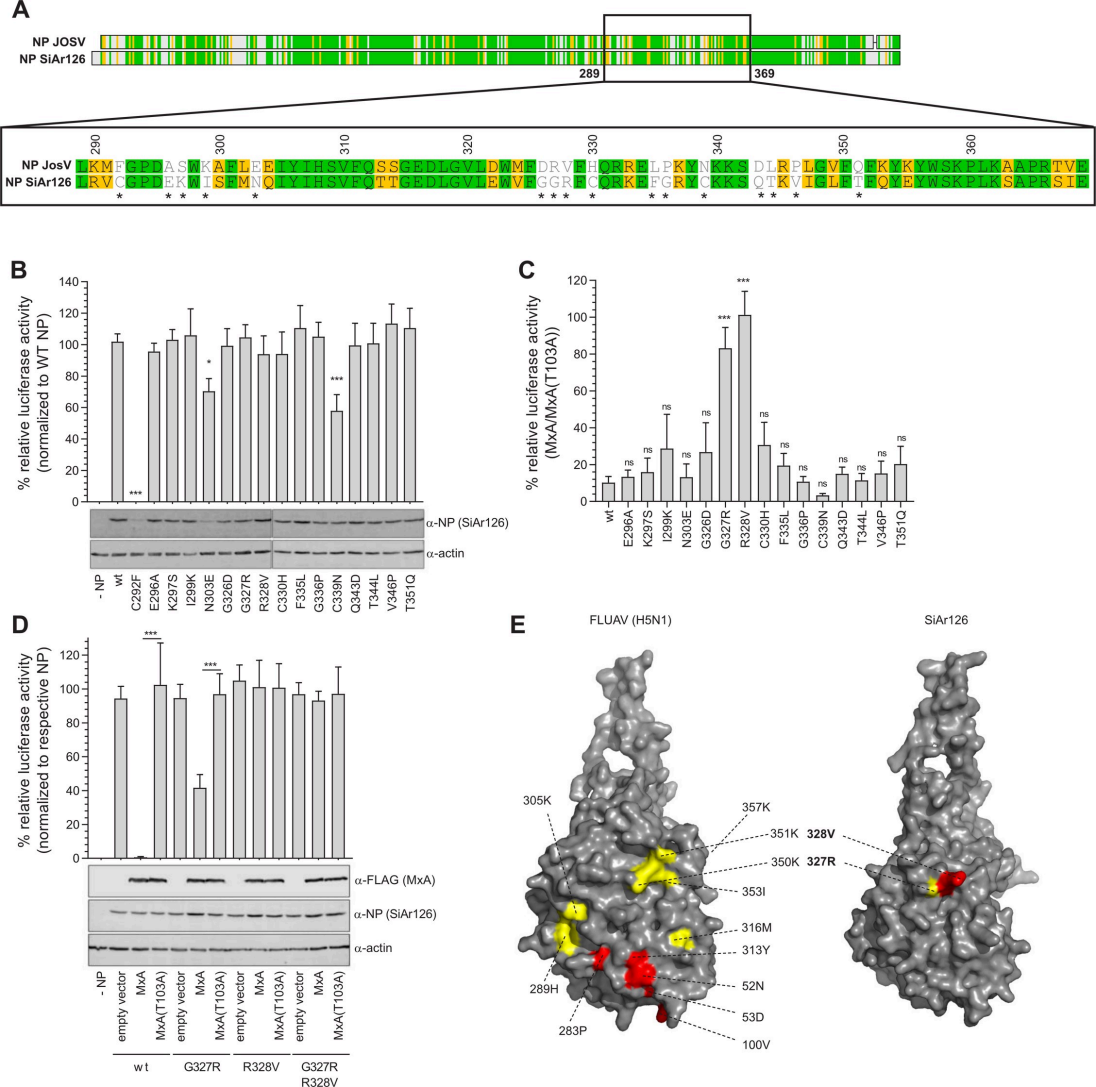
Positions G327 and R328 are responsible for MxA sensitivity. (A) Schematic representation of an amino acid alignment of JOSV and SiAr126 NP (green–identical, yellow–similar, white–non synonymous). Enlarged is the MxA sensitivity region corresponding to amino acids 289 to 370 of SiAr126 NP. Asterisks mark non-synonymous amino acid differences that could influence MxA sensitivity. (B-D) 293T cells were co-transfected with SiAr126 expression plasmids including 10 ng of PB1, PB2 and, PA, 50 ng of the individual NP mutants, 50 ng of pPol-I FF-Luc and 10 ng of RLuc. At 24 h after transfection the cells were lysed and firefly and Renilla luciferase activity determined. The firefly luciferase was normalized to the Renilla luciferase activity and the expression of NP, actin and MxA were controlled by Western blot. Significance was calculated with a one-way ANOVA (Tukey’s multiple comparison test, *p<0,05, ***p<0.001, ns–not significant). (B) The amino acid substitutions marked in (A) were introduced in SiAr126 NP and tested with the SiAr126 polymerase for activity in the absence of MxA. NP(WT) was set to 100% (mean ± SD, n = 3). (C) MxA sensitivity of the SiAr126 NP single mutants. 293T cells were transfected with the components of the SiAr126 system including the NP single mutants and 50 ng of MxA or MxA(T103A). Displayed is the ratio of the luciferase activity in the presence of MxA compared to MxA(T103A) (mean ± SD, n = 3). (D) Increased amounts of 300 ng MxA or MxA(T103A) were co-transfected with the SiAr126 system in the presence of 50 ng of NP expression plasmids coding for G327R, R328V or G327R/R328V. The empty vector control for the respective NP mutants was set to 100% (mean ± SD, n = 3). (E) Crystal structure of IAV (H5N1) NP (2q06.2.A) showing adaptive mutations responsible for MxA escape of pandemic IAVs [44] and a predicted SiAr126 NP structure (SWISS-MODEL; template H5N1 NP (2q06.2.A)) highlighting the positions 328V and 327R. Red–strong, yellow–weak influence on MxA sensitivity, for IAV according to Mänz et al. [44]. wt–wildtype.

Next, we screened this region for residues that impact MxA sensitivity based on an amino acid sequence alignment of both NP (Fig 3A). We introduced amino acid exchanges into the NPs of SiAr126 and JOSV at positions with major amino acid differences between both NPs. Introduction of the JOSV residues into SiAr126 NP did not lead to major differences in the SiAr126 polymerase activity, with the exception of the poorly expressed and inactive NP mutant C292F (Fig 3B). The inverse screen with SiAr126 residues into JOSV NP resulted in highly variable polymerase activities despite comparable expression levels and a large proportion of inactive mutants (S1 Fig). Therefore, only single mutants in the SiAr126 NP were used to analyze the MxA sensitivity. Most of the single mutations introduced into SiAr126 NP did not significantly alter MxA sensitivity. However, in case of the G327R and R328V substitutions in NP, MxA failed to inhibit the SiAr126 system (Fig 3C) (S2 Fig). Further analyses in the presence of increased amounts of MxA showed a partial escape of the viral polymerase activity from MxA inhibition by NP(G327R) and a complete escape by NP(R328V) or the double mutant (Fig 3D). This indicates that position 328 and to a lesser extend position 327 are critical for the MxA sensitivity of SiAr126.

Both positions are surface exposed in the predicted structures of monomeric SiAr126 NP (Fig 3E) and of higher order NP oligomers (S4 Fig). They are located in the NP body domain and correspond structurally (Fig 3E) and in a sequence alignment (S3 Fig) to positions K350 and K351 of IAV NP, respectively, that were described to influence the MxA sensitivity of pandemic IAV [44]. Remarkably, all THOV-like isolates except JOSV harbor an arginine at the position corresponding to 328 in SiAr126 NP (S3 Fig). To analyze how other amino acid residues at position 328 would influence the MxA sensitivity of SiAr126 NP, we introduced various exchanges representing charged (glutamic acid, lysine), polar (glutamine), large (tryptophan) and small (glycine) amino acids. Surprisingly, all exchanges at position 328 abolished the inhibition by MxA without grossly affecting the overall polymerase activity of SiAr126 (S5 Fig).

### Recombinant SiAr126 virus encoding NP(R328V) loses MxA sensitivity

**Fig 4:**
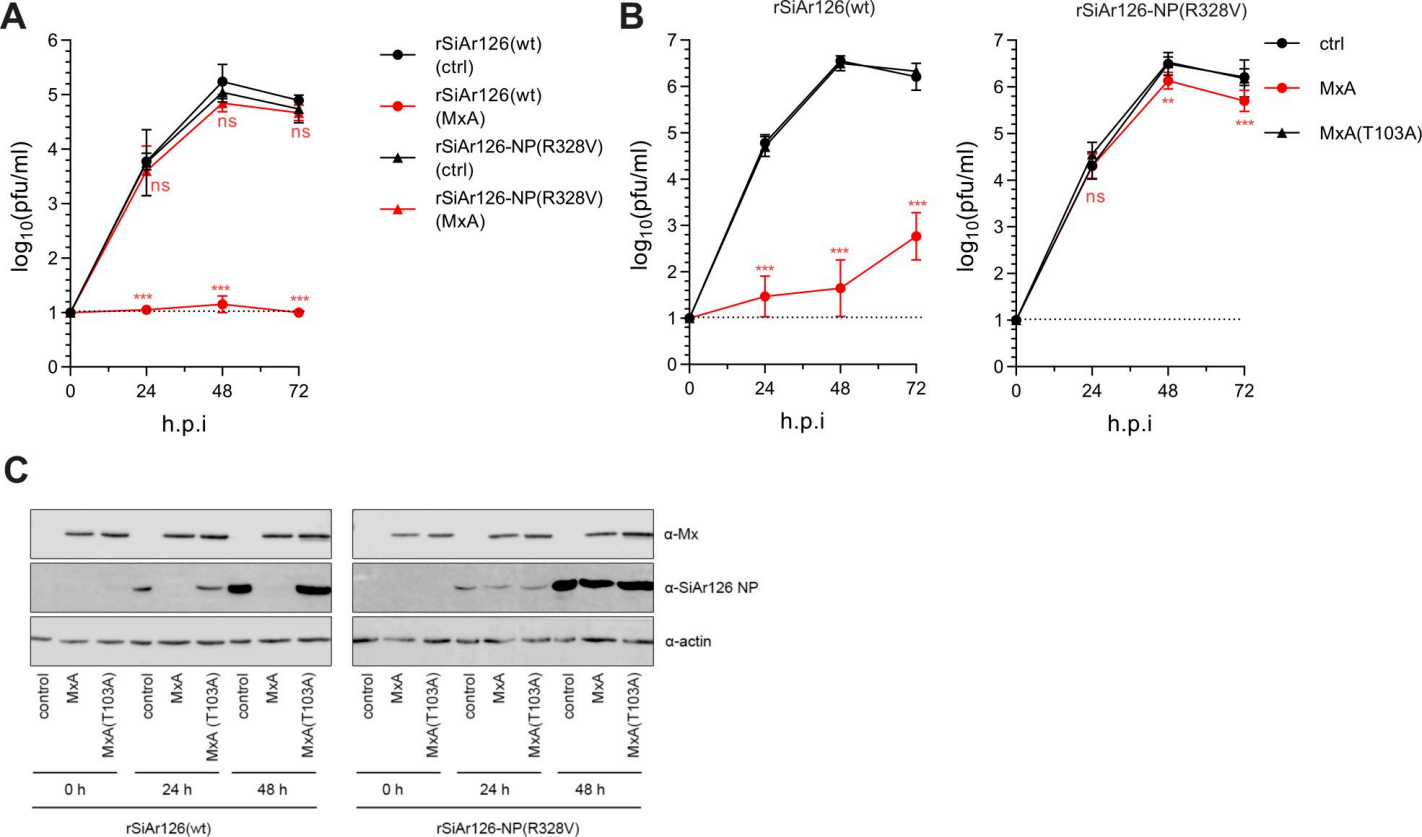
Recombinant SiAr126 NP(R328V) is MxA resistant. (A, B) Growth kinetics of rSiAr126(wt) or rSiAr126-NP(R328V) in the presence or absence of MxA. Vero-MxA or control Vero cells (A) or Huh7 cells stably expressing a control vector, MxA or MxA(T103A) (B) were infected with rSiAr126(wt) or rSiAr126-NP(R328V) (MOI 0.001). At the indicated time points the supernatants were harvested and the viral load determined by plaque assay (log-transformed values, mean ± SD, n = 3). Statistical analyses were performed with a two-way ANOVA (Tukey’s multiple comparison test, **p<0.01, ***p<0.001, ns–not significant). (C) As in (B) Huh7 cells were infected with rSiAr126(wt) or rSiAr126-NP(R328V) (MOI 0.01). At the indicated time points the cells were lysed and the expression of MxA, actin and viral NP controlled by Western blot.

Next, we evaluated the influence of NP exchanges G327R and R328V on viral fitness, using recombinant SiAr126 viruses. We successfully generated recombinant wildtype rSiAr126(wt) and rSiAr126-NP(R328V) but failed to rescue rSiAr126-NP(G327R) and rSiAr126-NP(G327R/R328V), despite multiple attempts. Introduction of the R328V mutation in rSiAr126-NP(R328V) was confirmed by sequencing. In the absence of MxA, the recombinant viruses grew to comparable titers in multicycle infections in Vero and Huh7 cells (Fig 4A and 4B), excluding any effects of the R328V exchange on virus replication. In the presence of MxA, rSiAr126(wt) replication was strongly attenuated in cultures stably over-expressing MxA whereas mutant rSiAr126-NP(R328V) was almost not affected (Fig 4A and 4B). Importantly, both viruses propagated normally on Huh7 cells stably expressing the antivirally inactive control MxA(T103A) (Fig 4B). Furthermore, we analyzed the production of viral NP in infected cells and confirmed that MxA had a strong suppressive effect on rSiAr126(wt) but not on rSiAr126-NP(R328V) replication (Fig 4C). These findings clearly demonstrate that the R328V virus gained MxA resistance by a single arginine to valine exchange.

**Fig 5:**
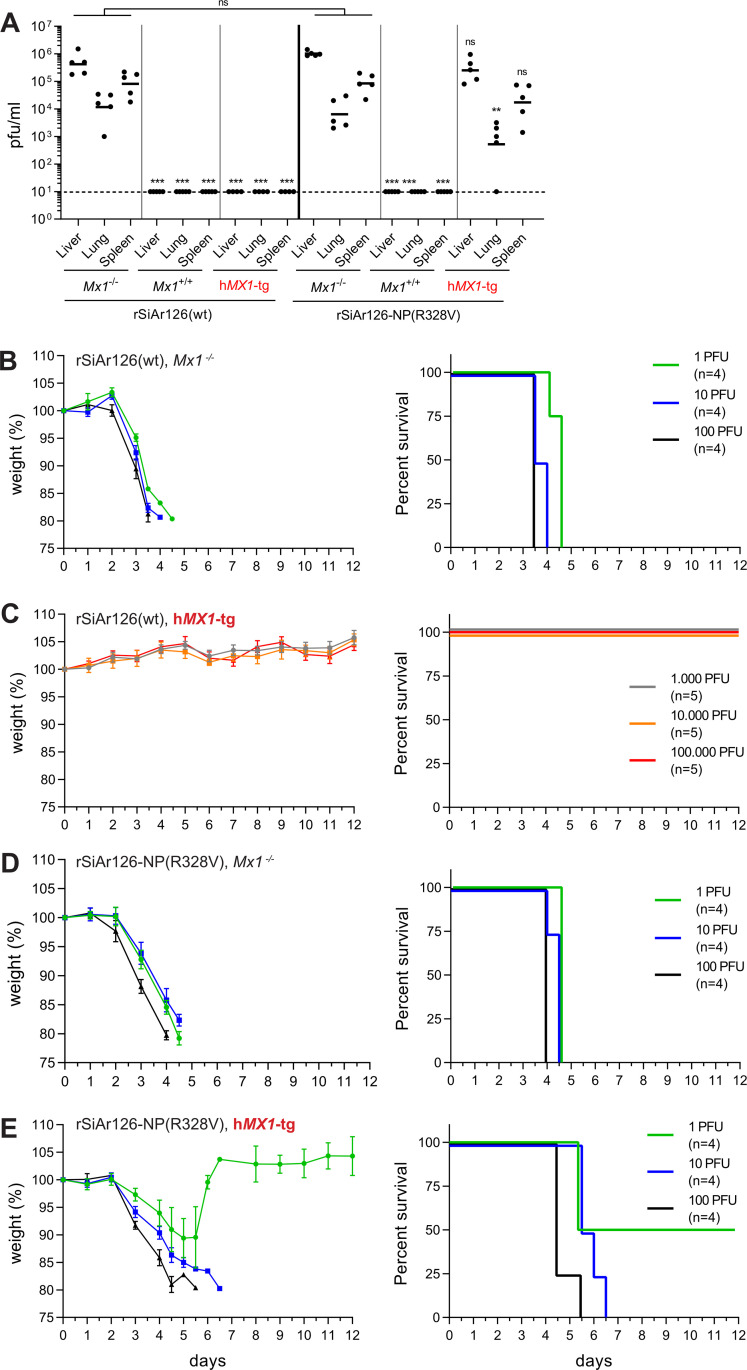
R328V leads to MxA escape *in vivo*. (A) C57BL/6 (*Mx1*^-/-^), congenic A2G-*Mx1* (*Mx1*^+/+^) and transgenic human *MX1* (h*MX1*-tg^+/+^) mice were infected with 100 pfu of rSiAr126(wt) or rSiAr126-NP(R328V) for 4 days. Organs were harvested and the viral load determined by plaque assay. Shown are the geometric means (n = 5). Statistical analyses were performed with a one-way ANOVA on log-transformed values (Tukey’s multiple comparison test, **p<0.01, ***p<0.001, ns–not significant) and displayed for each virus in comparison to the corresponding *Mx1*^-/-^ group. (B-E) Weight loss and survival of *Mx1*^-/-^ and h*MX1*-tg^+/+^ mice infected with rSiAr126(wt) (B, C) or rSiAr126-NP(R328V) (D, E). Mice were infected i.p. with the indicated inoculums (4 mice/group). Each day weight (mean ± SEM) and survival of the animals were monitored.

To study the replication and virulence of the recombinant viruses *in vivo*, C57BL/6 *Mx1*^-/-^ mice were infected with 100 pfu of rSiAr126(wt) or rSiAr126-NP(R328V). The viruses replicated to high organ titers at 4 days post infection (Fig 5A), comparable to the authentic SiAr126 isolate (Fig 1D). Accordingly, in control mice expressing murine *Mx1*^+/+^ replication of both viruses was completely blocked (Fig 5A). However, in h*MX1*-tg mice only infection with rSiAr126-NP(R328V) yielded progeny viruses in liver, lung and spleen whereas rSiAr126(wt) was suppressed to undetectable levels in all tissues (Fig 5A), indicating an efficient MxA escape of rSiAr126-NP(R328V) also *in vivo*.

Furthermore, we determined the survival of the animals upon infection with the recombinant viruses. Low dose infections with only one pfu of either virus caused a fatal disease in *Mx1*^-/-^ mice within four to five days (Fig 5B and 5D). As expected, the h*MX1*-tg mice were completely resistant to rSiAr126(wt) even at a high infection dose of up to 100,000 pfu. The animals did not show any signs of disease and all survived the infection (Fig 5C). In strong contrast, one pfu of rSiAr126-NP(R328V) caused a fatal disease in 50% of the h*MX1*-tg mice and ten pfu resulted in 100% fatality (Fig 5E). Interestingly, the course of the disease in the h*MX1*-tg animals was delayed by proximally one day compared to the *Mx1*^-/-^ mice (Fig 5D and 5E), indicating a residual MxA sensitivity of rSiAr126-NP(R328V). Together these results demonstrate that the NP mutation R328V confers almost complete MxA resistance in cell culture as well as *in vivo* without any obvious cost in viral fitness.

### NP variant R328V escapes recognition by MxA

**Fig 6:**
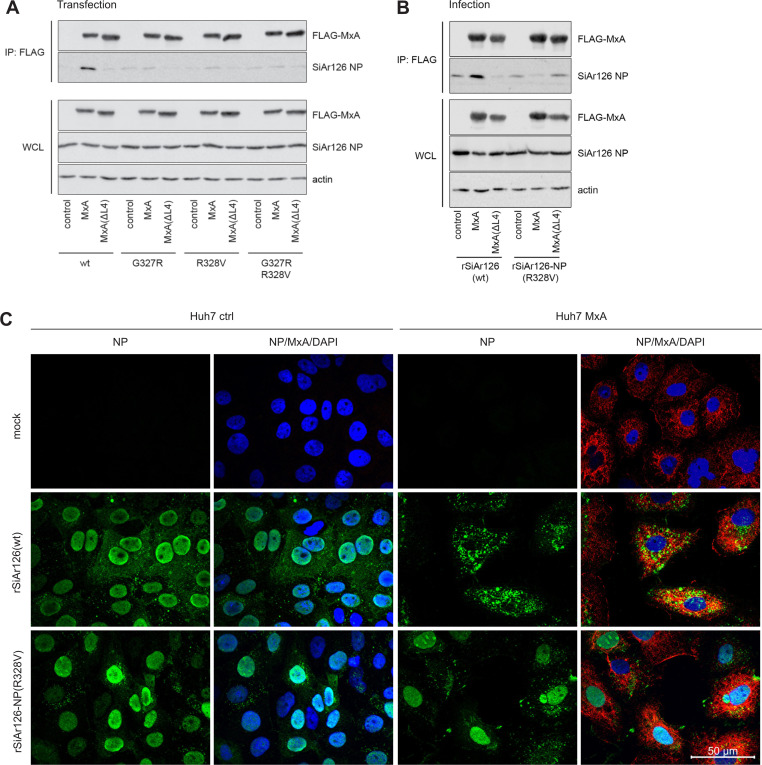
Loss of NP recognition by MxA is responsible for MxA escape. (A) Co-immunoprecipitation (CoIP) of NP with MxA from transfected cells. 293T cells were co-transfected with the components of the SiAr126 polymerase system, including 100 ng of PB1, PB2, PA, 500 ng of the individual NP mutants, 500 ng of pPol-I FF-Luc and 700 ng of FLAG-MxA or FLAG-MxA(ΔL4). (B) CoIP of NP with MxA from rSiAr126(wt) and rSiAr126-NP(R328V) infected cells. 293T cells were transfected with 1,000 ng of FLAG-MxA or FLAG-MxA(ΔL4) and after 24 h infected with the recombinant viruses (MOI 10) for 24 h. (A and B) Cells were harvested and Flag-MxA was precipitated via anti-FLAG coupled beads from the lysates. MxA, viral NP and actin were detected in whole cell lysates (WCL) and the MxA precipitates (IP: FLAG) via Western blot. Representative Western blots of 3 individual experiments are shown. (C) Immunofluorescence pictures of Huh7 ctrl and MxA(wt)-expressing cells infected with rSiAr126(wt) and rSiAr126-NP(R328V). Huh7 ctrl and MxA cells were infected for 6 h (MOI 50), fixed on coverslips and stained for viral NP (green), MxA (red) and for the nucleus (DAPI, blue). Pictures were taken with an Apotome. wt–wildtype.

The antiviral effect of MxA is based on the interaction of MxA with the viral NP in the cytoplasm leading to impaired nuclear import of the incoming vRNPs [26,45]. To analyze the consequences of the escape mutations in NP, we first studied the binding of MxA to NP in co-immunoprecipitation assays [26,27]. 293T cells were mock transfected or transfected with expression plasmids encoding Flag-tagged MxA wildtype or an MxA mutant (MxA(ΔL4)) lacking the key interface recognizing vRNPs of THOV-like viruses [27]. The cells were co-transfected with the components of the SiAr126 polymerase reconstitution system and the expression plasmids for wildtype or mutant NP(G327R), NP(R328V) or double mutant NP(G327R/R328V). Subsequently, MxA was precipitated from the cell lysates using a Flag-tag specific antibody. Western blot analysis of the precipitates showed that wildtype NP but none of the three NP mutants co-precipitated with MxA despite comparable expression levels in the whole cell lysates (Fig 6A). All tested NPs did not co-precipitate with MxA(ΔL4), as expected. Moreover, we infected MxA-transfected 293T cells with rSiAr126(wt) or rSiAr126-NP(R328V) and performed Flag-specific immunoprecipitation after 24 h. Wildtype MxA clearly co-precipitated NP of rSiAr126(wt), whereas there was only a faint background signal detectable in the precipitate from cells infected with rSiAr126-NP(R328V) (Fig 6B). To evaluate the consequences of this MxA/NP interaction on the viral replication cycle, we infected control Huh7 cells or cells stably expressing wildtype MxA for 6 h. Immunofluorescence analysis of the control cells revealed a clear nuclear NP accumulation after infection with both viruses. In infected, MxA-overexpressing cells, the NP of rSiAr126(wt) was exclusively detected in the cytoplasm, whereas the NP signal of rSiAr126-NP(R328V) infected cells accumulated in the nucleus unperturbed by co-expressed MxA (Fig 6C). This suggests that THOV-like viruses can escape MxA restriction by evading the NP recognition of MxA and are therefore able to efficiently translocate to the nucleus and initiate viral replication.

### NP mutants G327R and R328V escape a diversity of MX1 variants and orthologs

**Fig 7:**
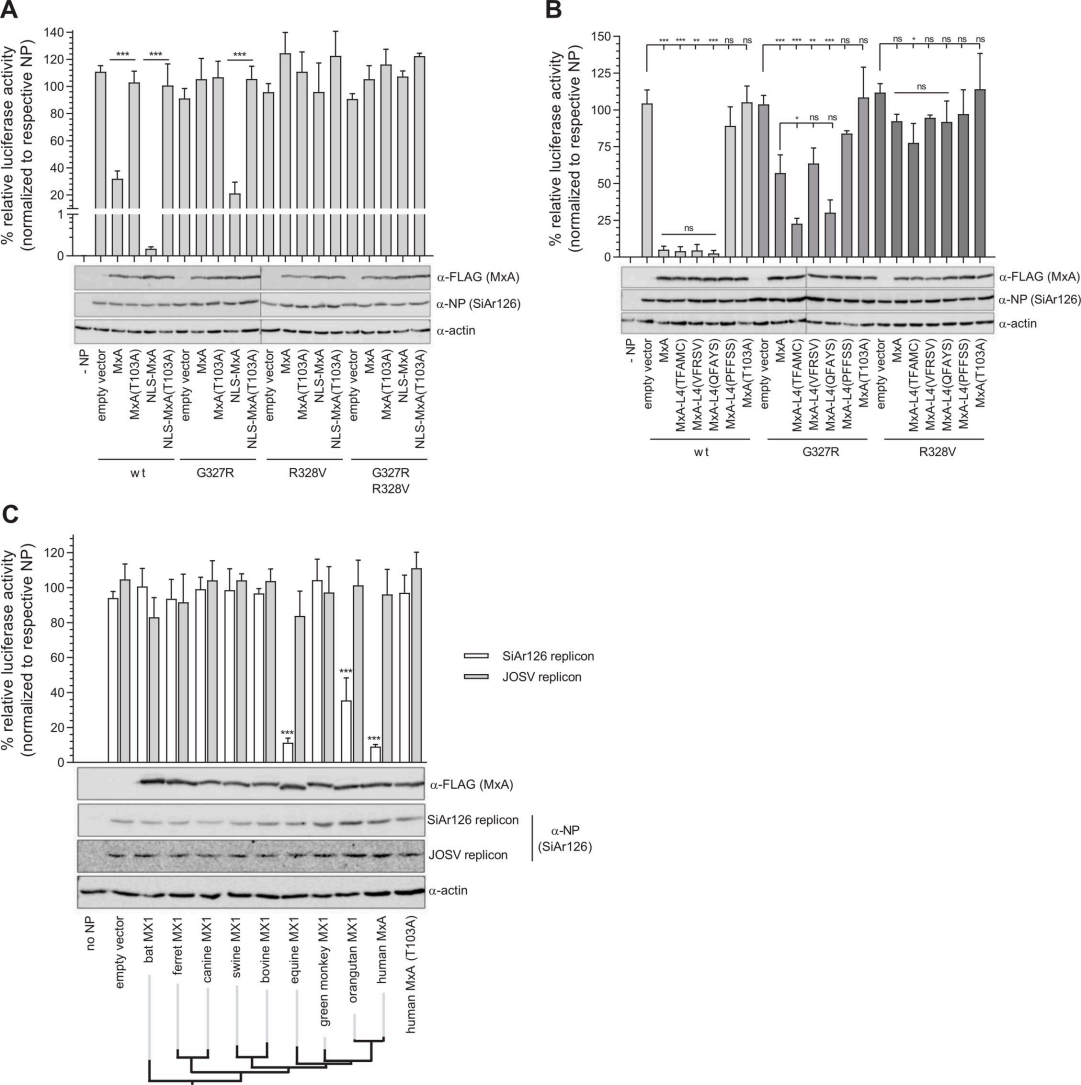
SiAr126 NP mutants escape restriction by different MX1 variants. (A-C) 293T cells were co-transfected with the components of the polymerase reconstitution system, including 10 ng of PB1, PB2, PA, 50 ng of NP, 50 ng of pPol-I FF-Luc and 10 ng of RLuc. At 24 h after transfection the cells were lysed and the firefly and Renilla luciferase activity determined. Firefly luciferase activity was normalized to the Renilla luciferase activity and the expression of NP, actin and MxA was controlled by Western blot. Significance was calculated with a one-way ANOVA (Tukey’s multiple comparison test, ***p<0.001, ns–not significant). (A) 293T cells were transfected with the components of the SiAr126 system including 50 ng of wildtype NP, NP(G327R), NP(R328V) or NP(G327R/R328V). To analyze the influence of nuclear MxA, 300 ng of MxA, MxA(T103A), NLS-MxA or NLS-MxA(T103A) were co-transfected. The empty vector control for the respective NP mutants was set to 100% (mean ± SD, n = 3). (B) 293T cells were transfected with the components of the SiA126 system including 50 ng of wildtype NP, NP(G327R) and NP(R328V). To analyze the influence of MxA super-restrictors, 600 ng MxA, MxA(T103A) or MxA constructs with mutations in loop 4 (L4) were co-transfected. The empty vector control for the respective NP mutants was set to 100% (mean ± SD, n = 3). (C) 293T cells were transfected with the components of the SiAr126 or JOSV system. Different mammalian MX1 proteins were tested by transfecting 50 ng of bat MX1 (*Eidolon helvum*), ferret MX1, canine MX1, swine MX1, bovine MX1 with a repaired ORF, equine MX1, African green monkey MX1, orangutan MX1, human MxA, and MxA(T103A). The empty vector control for the respective polymerase systems was set to 100% (mean ± SD, n = 3). wt–wildtype.

Next, we explored the capacity of the SiAr126 NP mutants to escape MxA restriction in different subcellular compartments. Human MxA has been shown to restrict IAV replication even if it is relocalized from the cytoplasm to the nucleus via a foreign nuclear localization signal (NLS-MxA) [46]. Expression of NLS-MxA in the wildtype SiAr126 polymerase reconstitution assay diminished reporter gene expression (Fig 7A), while the respective inactive controls, MxA(T103A) and NLS-MxA(T103A), did not show any inhibitory effects. Interestingly, the inhibition of the viral polymerase activity by NLS-MxA was about 10-times stronger in comparison to cytoplasmic wildtype MxA. The reconstitution system combined with NP(G327R) showed a reduced antiviral effect of wildtype MxA and NLS-MxA. Remarkably, SiAr126 NP(R328V) and NP(G327R/R328V) completely escaped NLS-MxA restriction (Fig 7A), indicating that their MxA escape also happens in the nuclear compartment.

Recently, an evolution-guided approach identified MxA variants with an increased antiviral activity against SiAr126 [47]. These super-restrictors (SR) vary at sites in the L4 that have been reported to be under positive selection [43,47]. We tested if NP mutations G327R and R328V would be able to escape these MxA SRs (Fig 7B). In the presence of wildtype NP the MxA SRs but not the non-restrictor (NR) control, MxA-L4(PFFSS), potently inhibited the polymerase activity. The enhanced potency of at least two of the MxA SRs became apparent when NP(G327R) was used. The viral polymerase activity was apparently more strongly restricted by the two SRs MxA-L4(TFAMC) and MxA-L4(QFAYS) compared to wildtype MxA (Fig 7B). However, none of the SRs were able to reduce the polymerase activity in the presence of NP(R328V) (Fig 7B), highlighting the exceptionally efficient MxA escape of this NP mutant.

The *in vivo* experiments (Fig 5A) demonstrated that the NP(R328V) mutation allowed escape specifically from human MxA but not from mouse MX1. To evaluate this astonishing specificity, we tested a broader collection of MX1 proteins from different species which have been shown to be antivirally active against influenza A viruses. We cloned the cDNA of eight different mammalian MX1 homologs from bat [48], ferret [49], dog [50], swine [44], cow [51], horse [52], orangutan, and African green monkey [43] into identical expression vectors and determined their activity in the polymerase reconstitution assays of SiAr126 and JOSV. Interestingly, the SiAr126 activity was only restricted by the co-expression of human MxA, the humanoid orangutan MX1 and equine MX1. However, none of the different MX1 proteins were able to block the JOSV polymerase activity. Collectively, our data demonstrate that the NP(R328V) mutation allows escape from nuclear MxA and even from MxA super-restrictors. Moreover, JOSV can likely escape multiple MX1 orthologs present in diverse mammalian species.

## Discussion

Tick-transmitted thogotoviruses have to cope with the innate immune response of the mammalian host to initiate successful infection. Here, we investigated to what extent thogotoviruses of different geographical regions are capable of overcoming the antiviral action of the human MxA protein, a known IFN-induced antiviral restriction factor [24,39,53,54]. We found that thogotoviruses differed in their MxA sensitivity according to their phylogenetic affiliation because DHOV-like thogotoviruses were MxA resistant and THOV-like viruses were highly sensitive. In contrast, JOSV, a THOV-like isolate from Nigeria [55], escaped MxA restriction, due to two amino acid exchanges in the viral NP (positions 327 and 328 in SiAr126 NP) that are conserved among MxA-sensitive THOV-like viruses. Remarkably, we found that the introduction of one single amino acid exchange (R328V) into the NP of SiAr126 resulted in full MxA escape without any viral fitness loss. Our results clearly demonstrate that human MxA is a key factor restricting THOV-like viruses *in vivo* and that these viruses can escape by a single amino acid exchange in NP.

**Fig 8:**
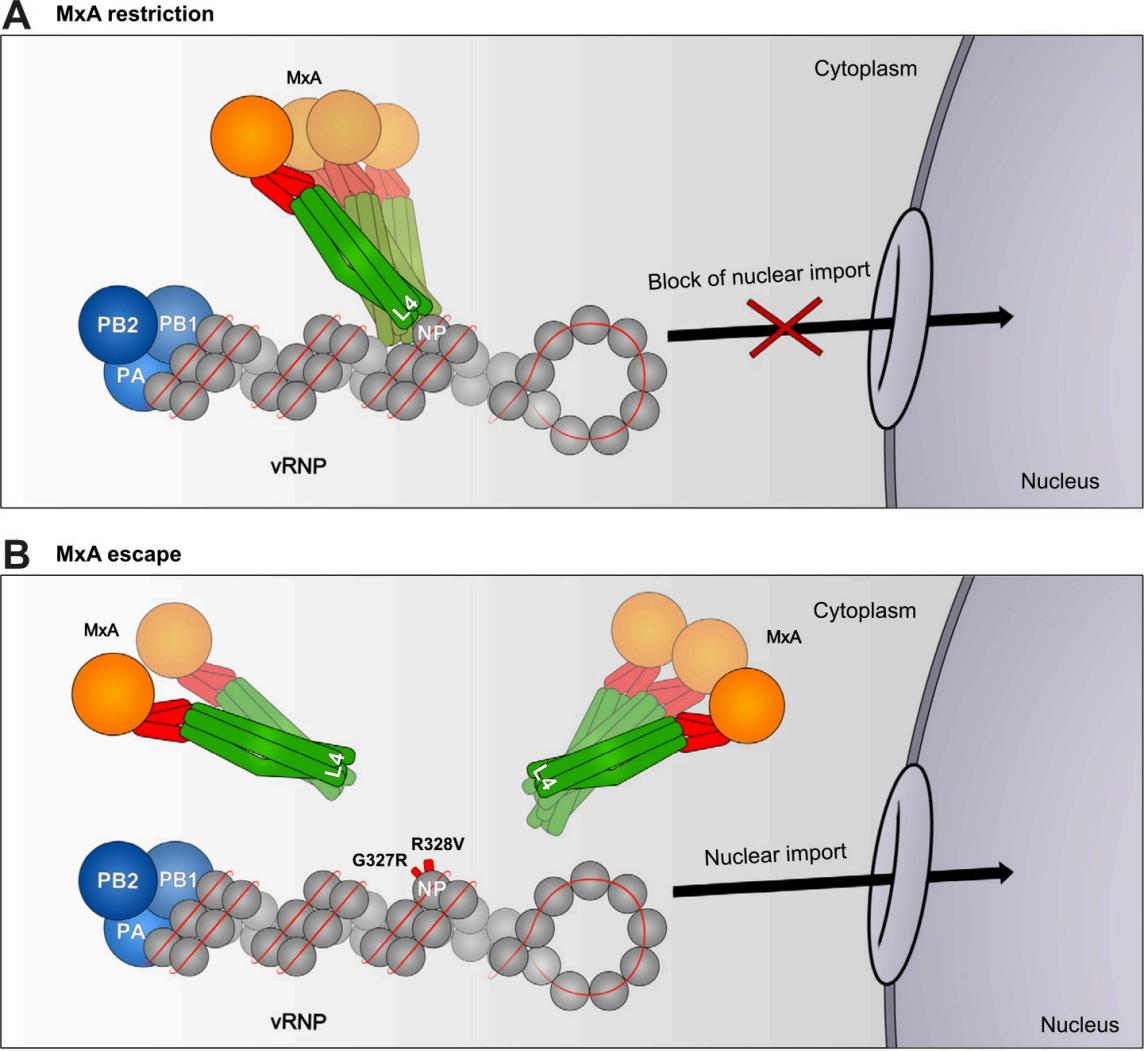
Model for the MxA escape by thogotoviruses. (A) Cytoplasmic MxA mono- and oligomers interact (direct or indirect) via their unstructured loop 4 (L4) with incoming vRNPs by recognizing surface exposed residues in NP leading to their retention in the cytoplasm. (B) Positions G327 and R328 are crucial for the MxA-NP interaction. Mutations at these positions lead to the loss of this interaction. Therefore incoming vRNPs can escape MxA recognition, translocate into the nucleus and initiate viral transcription and replication.

MxA is a large GTPase of the dynamin superfamily and consists of a globular GTPase domain, a bundle signaling element and an alpha-helical stalk [56,57]. A disordered loop (L4) at the tip of the stalk provides an interface for recognition of various viral targets (Fig 8A) [27,43]. MxA has been shown to recognize the NP of incoming vRNPs in SiAr126-infected cells and prevents their nuclear import (Fig 8A) thereby efficiently preventing early transcriptional activity that would initiate viral replication [28,39]. This scenario has also been postulated for the antiviral effect of MxA against IAV [45,58]. The present results are compatible with the proposed model of antiviral action as MxA did not co-precipitate with the modified NP(G327R), NP(R328V) and NP(G327R/R328V) and was unable to prevent the nuclear import of recombinant SiAr126-NP(R328V) in infected cells (Fig 8B).

The R328V substitution in SiAr126 NP did not affect the growth capacity of the recombinant virus, both in cell culture and *in vivo*. This was unexpected because, in the case of IAV, MxA escape-associated substitutions in NP usually result in virus attenuation, possibly due to misfolding of NP [59] and/or defective nuclear vRNP import [60]. Obviously, position 328 is not critical for viral growth but hypercritical for MxA sensitivity. The surface exposed residues 327 and 328 both influence MxA sensitivity and are located in the predicted main body domain of NP (Fig 3E). Remarkably, MX sensitivity-determining residues of IAVs reside in an analogous region of the viral NP [44]. NP residues 350K, 351K and 353I that contribute to MX-insensitivity of A/Hamburg/4/2009 (pH1N1) correspond to positions 327 and 328 of SiAr126 NP (Fig 3E) (S3 Fig). Interestingly, the valine at position 328 in JOSV NP is also conserved in the NP sequences of MxA-resistant DHOV-like viruses, except for BRBV NP, which encodes a glutamic acid at this position. In future studies, we will clarify the contribution of this NP region for the MxA escape of DHOV-like viruses. This region is distinct from the RNA-binding and polymerase interaction domains of NP [61,62], and also distinct from the domains responsible for NP self-assembly [63,64]. Modeling the two NP escape positions into a putative structure of THOV vRNPs (S4B Fig), guided by the 3D structure of IAV vRNPs [64,65], indicates that these two critical surface-exposed residues are accessible by MxA even upon assembly of the NPs into functional vRNPs. Multiple repetitive NP contact sites presumably stabilize the MxA-vRNP interactions. Formation of stable MxA-vRNP complexes may help to retain the nucleocapsids in the cytoplasm of the infected cells, as reported for IAV [45,58]. Despite much effort, however, such complexes could so far not be visualized and the decoration of vRNPs by MxA remains to be demonstrated. Alternatively, MxA might shield the vRNPs from interacting with other cellular proteins, such as nuclear import factors. However, MxA does most likely not target the NLS of SiAr126 NP (aa 179–193) since the putative MxA interaction site (aa 289–370) appears not to be in close proximity, as revealed by the predicted 3D structure (S3 and S4 Figs).

An evolution-guided screen of primate MxA proteins revealed that the extended loop L4 of human MxA is a major determinant of antiviral specificity against THOV and IAV [27,43]. Surprisingly, random mutagenesis of critical positions within this L4 loop led to amino acid combinations that exhibited increased super-restrictor antiviral activity [47]. As shown here, these MxA SRs had enhanced potency to block wildtype SiAr126 and mutant SiAr126- NP(G327R) in the polymerase reconstitution system. In sharp contrast, inhibition of SiAr126-NP(R328V) by these SRs was minimal, demonstrating the efficiency of MxA escape caused by the single amino acid exchange in NP.

The available sequence information indicates that the MxA sensitivity determining residues are conserved in most THOV-like isolates and that the acquisition of MxA escape mutations is a rare event (S3 Fig). The evolutionary advantage of gaining resistance to MxA is presently unknown and the influence of MxA escape mutations on the replication capacity in ticks was not addressed in the present study. The escape mutations may have been gained during JOSV adaptation to a new host, allowing a certain degree of immune evasion. Since a specific spectrum of mammalian MX1 proteins might have shaped JOSV NP, we compared the MX1 susceptibility of JOSV with that of SiAr126 using MX1 proteins from nine different species that represent roughly 100 million years of evolutionary divergence [66]. In all cases, MX1 expression did not reduce the JOSV polymerase activity (Fig 7C), demonstrating that JOSV had gained resistance to a broad range of mammalian MX1 proteins. JOSV was initially isolated from ticks feeding on cattle in Central Africa [32,67]. Thus, the selection pressure of bovine MX1 might have led to the introduction of the two MX1 escape mutations in NP. In analogy, the evolution of the MX1 escape of IAV is based on a stepwise accumulation of critical amino acid changes in NP. For example, IAVs circulating in swine accumulated MX1 escape mutations in the presence of porcine MX1 that finally resulted in the zoonotic transmission of an MxA-resistant pandemic pH1N1 in 2009 [44].

Despite their ability to escape the antiviral effect of MxA, thogotoviruses remain sensitive to the murine paralog MX1. It has been shown that thogotovirus infections are highly lethal for *Mx1*-negative mice [6,68]. In contrast, mice expressing a functional *Mx1* gene survive a thogotovirus infection [69,70], yet are still able to infect co-feeding ticks, even in the absence of detectable viral replication [71]. It is therefore conceivable that the antiviral effect of murine MX1 has an evolutionary advantage for thogotoviruses, because they can be horizontally transmitted within a tick population without affecting the fitness of the infected rodents. The unique role of murine MX1 is further demonstrated by its accumulation in the cell nucleus [72,73]. It has been proposed that the antiviral mechanism of murine MX1 differs from that of cytoplasmic human MxA [74,75]. Whereas MxA targets the viral NP and blocks the nuclear import of vRNPs, mouse MX1 restricts viral replication in the cell nucleus by targeting NP and the PB2 polymerase subunit, subsequently disturbing PB2-NP interaction [76]. Accordingly, the antiviral effect of murine MX1 can be bypassed by overexpression of the viral PB2 subunit [77]. Because of these additional constraints, it is feasible that the here identified MxA escape mutations are not sufficient to bypass murine MX1.

Thogotoviruses show a broad host range and serological studies provide evidences for sporadic zoonotic contacts [6–8]. However, we show that human MxA represents a potent antiviral barrier against THOV-like viruses and might protect humans from zoonotic infections. Some thogotoviruses, however, manage to escape MxA restriction and might become the source of severe infections. The MxA escape mutations described here could be used to predict the pathogenic potential of newly identified thogotovirus isolates and might help to estimate their zoonotic threat.

## Materials and methods

### Ethical statement

All animals were handled in compliance with regulations of the German animal welfare law. The protocols were approved by the local animal care committee (Regierungspraesidium Freiburg, Az. 35–9185.81/G-15/127).

### Cell lines, culture and interferon treatment

Human lung epithelial A549 cells (ATCC CCL-185), human hepatoma Huh7 cells [78], human cervix carcinoma HeLa cells (ATCC CCL-2), Syrian golden hamster kidney cells BHK-21 (ATCC CCL-10), African green monkey kidney Vero cells (ATCC CCL-81), Vero MxA cells stably overexpressing *MX1* cDNA [39] and human embryonic kidney 293T cells (ATCC CRL-3216) were cultivated in Dulbecco's Modified Eagle Medium (DMEM) supplemented with 10% fetal calf serum (FCS) at 37°C and 5% CO_2_. Huh7 cells and A549 were treated with recombinant human IFN-α2a (PBL assay science) or IFNαB/D [42] for 24 h before Western blot analysis.

### Generation of stable cell lines

Huh7 cells expressing human MxA or MxA(T103A) were generated by lentiviral transduction. The MX1 cDNA was cloned into the lentiviral pLVX-Puro expression vector (Clontech). Lentiviruses were produced in 293T cells by co-transfecting pLVX-MxA or pLVX-MxA(T103A), pCMVR8.91 and pMDG(VSV-G) as previously described [79]. 72h post transfection, the supernatant was harvested. Huh7 cells were infected with the lentivirus and selection with 2 μg/ml Puromycin was started 48 h post transduction.

### Viruses, infection and plaque forming assay

Thogotovirus isolates used in the present study: THOV HI-Kamigano-25 [38], THOV AfricAII [31], THOV PoTi503 [34], THOV SiAr126 [33], JOSV [32], Oz virus[37], BRBV [18], DHOV India [4], BTKV [36] and DHOV PoTi461 [35].

All infections with BRBV or rSiAr126 mutants with reduced MxA sensitivity were performed under BSL3 conditions because of the unknown health risk of these pathogens.

Virus infections were performed by washing the cells once with PBS and infecting them at a certain multiplicity with virus diluted in Opti-MEM for 1.5 h at RT. After the infection, fresh medium containing DMEM with 1% FCS and 20 mM HEPES was supplied.

BHK cells were infected at a multiplicity of infection (moi) of 0.001 to produce virus stocks. The supernatant were harvested 48 to 72 h.p.i at a medium CPE and centrifuged at 3,000 rpm for 5 min. Virus stocks were aliquoted and stored at -80°C. In case higher virus titers of rSiAr126 were needed for co-immunoprecipitation (CoIP) and immunofluorescence analysis, virus supernatants were subjected to ultracentrifugation (100,000 x g, 90 min, SW-32 rotor) and the pellets re-suspended in PBS.

For growth kinetics the cells were infected at an moi of 0.001 or 0.01 (for Western blot time kinetic) in Opti-MEM for 1.5 h at RT. Virus-containing supernatants were stored at -80°C and viral titers were determined by plaque assay on Vero cells.

A plaque assay was performed by serial dilutions of the samples in PBS and incubation of the dilutions on Vero monolayers for 1.5 h at RT. The infection inoculums were removed and the cells overlaid with a medium containing 0.6% oxoid agar solution and 1% FCS. After incubation for 72 h at 37°C, cells were fixed with formaldehyde (3.7% in PBS) and stained with 0.1% crystal violet.

### RNA Extraction and cDNA cloning

To clone the components of the JOSV polymerase reconstitution system, Vero cells were infected with JOSV (moi = 0.25) for 24 h. RNA was isolated using the NucleoSpin RNA Kit (MACHEREY-NAGEL) according to the manufacturers’ protocol. Total RNA (1 μg) was reverse transcribed using the RevertAid H Minus First Strand cDNA Synthesis Kit (ThermoFisher Scientific). The ORF of JOSV PB1, PB2, PA and NP were amplified by PCR (KOD hot start polymerase, Novagen®) and cloned into the pCAGGS expression vector.

Chimeric NP constructs of JOSV and SiAr126 NP were constructed by determining highly conserved regions between both NP sequences (S3 Fig). The JOSV or SiAr126 NP parts were individually amplified by PCR. In a second PCR the fragments were merged due to overlapping sequences. For in vitro mutagenesis a two-step PCR was performed, in which the target gene was first amplified in two parts with internal primers harboring the desired nucleotide substitution. These two parts of the insert were used as templates in a subsequent second PCR to generate the full-length target sequence. The PCR products were digested and ligated into the digested and dephosphorylated expression vector via a T4 DNA ligase.

### Polymerase reconstitution assay

To reconstitute the polymerase activity of SiAr126 or JOSV, 293T cells were cultured in 12 well plates for 24 h and then co-transfected (JetPEI, Polyplus) with 10 ng of pCAGGS expression plasmids encoding the polymerase subunits PB2, PB1, PA and 50 ng of NP plasmids as previously described [48]. In addition, 50 ng of an artificial viral minigenomes encoding firefly luciferase in negative sense orientation flanked by viral non-coding regions (Pol-I-FF-Luc) and 10 ng of a plasmid coding for a Renilla luciferase under the constitutive SV40 promotor (SV40-RL) were added. To determine the effect of MX1 proteins on the activity of the viral polymerase, various MxA expression constructs or different mammalian MX1 homologs were co-transfected. At 24 h post transfection the firefly and Renilla luciferase activities were measured (Dual-Luciferase Reporter Kit, Promega). Firefly luciferase activity was normalized to Renilla luciferase activity. Western blot analyses were performed with specific antibodies directed against FLAG (Sigma-Aldrich) or MxA [80], SiAr126 NP [26] and α-actin (Sigma-Aldrich). JOSV NP was detected using post infectious polyclonal mouse anti-serum.

### Immunoblotting

The cell lysates of the polymerase reconstitution assay were mixed with SDS sample buffer. For all other Western blots, the cells were lysed directly with a 1:1 mix of T-PER tissue protein extraction reagent and SDS sample buffer (Thermo Fisher). Following denaturation at 95°C for 5 mins the samples were separated by gel electrophoresis (12% acrylamide). The proteins were wet blotted at 240 mA for 2 h onto a PVDF membrane (Merck). The membrane was blocked for 1 h with blocking buffer (0.1% Tween-20, 5% milk powder in PBS). Next the membrane was incubated with the primary antibody (diluted in blocking buffer) for 1 h at RT and washed three times for 10 min with washing buffer (0.1% Tween-20 in PBS). After incubation with the secondary fluorescent labeled antibody (LI-COR, diluted in blocking buffer) for 1 h at RT, the membrane was washed 4 times for 5 min with washing buffer. The fluorescent signals were detected using the ODYSSEY®Fc (Licor). Primary antibodies that were used for Western blot: anti-SiAr126 NP (rabbit, polyclonal [26]), anti-JOSV (mouse, polyclonal), anti-MxA (M143, mouse, monoclonal [80]), anti-FLAG M2 (mouse, monoclonal, Sigma-Aldrich) and anti-β-actin (rabbit, polyclonal, Abcam).

### Fluorescence microscopy

For immunofluorescence microscopy analysis the cells were seeded onto cover slips and infected. The cells were fixed in paraformaldehyde (4% in PBS) for 15 min at RT and washed with PBS. Afterwards the cells were permeabilized with Triton-X (0.5% in PBS). After blocking for 1 h with blocking buffer (PBS with 1% BSA and +0,1% Tween 20) the cover slips were incubated with the primary antibody for 1 h at RT. Unspecific binding was reduced by washing five times with PBS. The secondary antibody was incubated in the dark at RT for 1 h. After washing once with PBS, the cells were stained with DAPI (0.3 μM in PBS) for 10 min, washed again three times with PBS and mounted onto microscope slides using FluorSave. Pictures were taken with an Apotome (Carl ZEISS AG). Primary antibodies that were used for immunofluorescence: anti-SiAr126 NP (mouse, monoclonal, 3D11, kindly provided by A. R. Filipe, Centre for Zoonoses Research, National Institute of Health, Lisbon, Portugal) and anti-MxA (rabbit, polyclonal [26]).

### Generation of recombinant viruses

Recombinant viruses were generated by transfecting (Lipofectamine 2000) a 1:1 co-culture of Vero and 293T cells (6 well format) with ambisense pHW2000 plasmids coding for all 6 segments of SiAr126 (500 ng/each) [81]. At 96 h after transfection the supernatant was harvested and a plaque assay performed on Vero cells. Virus stocks of the rSiAr126(wt) or rSiAr126-NP(R328V) virus were produced by transferring single plaques to fresh BHK-21 cells. The NP segment was sequenced from the cDNA of infected cells to control for the presence of the introduced mutation.

### Co-immunoprecipitation (CoIP)

CoIP studies were performed using 293T seeded in 6-wells and transfected with pCAGGS expression plasmids: 100 ng PB1, PB2, PA, 500 ng SiAr126 NP, 500 ng pPol-I FF-Luc and 700 ng FLAG-MxA or FLAG-MxA(ΔL4). Alternatively, the cells were transfected with 1,000 ng FLAG-MxA or FLAG-MxA(ΔL4) and after 24 h infected with rSiAr126 at a moi of 10 for 24 h. The cells were harvested with a cell scraper, centrifuged (5 min, 1,000 rpm, 4°C) washed once with PBS and again centrifuged (5 min, 3,000 rpm, 4°C). The cell pellet was lysed for 15 min on ice in 300 μl CoIP buffer (50 mM Tris pH 8, 150 mM NaCl, 1 mM EDTA pH 8, 0.5% NP40) containing protease inhibitors (Roche) and subsequently centrifuged (10 min, 13,000 rpm, 4°C). 60 μl supernatant was separately stored for the expression control. The remaining supernatant was incubated with 30 μl ANTI-FLAG® M1 Agarose Affinity Gel (Sigma) at 4°C for 2 h. The beads were washed five times with 500 μl CoIP buffer (2 min, 500 rpm, 4°C) and the precipitate eluted in 1x SDS sample buffer at 95°C for 5 min. The CoIP samples were then subjected to Western blot analysis.

### Animal experiments

Wildtype C57BL/6 mice (*Mx1*^-/-^) have a natural defect in Mx1 expression [40] and were purchased from Janvier Labs (France). Congenic mice with a reconstituted *Mx1* locus (B6.A2G-*Mx1*^+/+^) [41] and mice transgenic for the human MxA (B6.h*MX1*-tg, homozygous) [29] were backcrossed to C57BL/6 animals and bred in house. The h*MX1*-tg mice carry a bacterial artificial chromosome including the entire human *MX* locus. Therefore h*MX1* is expressed under the control of its authentic IFN-inducible promoter [29]. The animals were handled in accordance with guidelines of the Federation for Laboratory Animal Science Associations and the national animal welfare body. Animal experiments were performed in compliance with the German animal protection law and approved by the local animal welfare committee (Regierungspraesidium Freiburg, permit G-15/127). All experiments were performed with age matched animals (7 to 10 weeks old mice). Animals were infected intraperitoneally (i.p.) with SiAr126 or JOSV diluted in 100 μl PBS. The animals were killed by cervical dislocation at the indicated time points. To determine survival of the animals upon infection, their weight was monitored daily. If the weight loss was greater than 25% or the mice showed severe disease symptoms (ruffed fur, lethargy and hunched posture) the mice were sacrificed. For viral titers of liver, lung, and spleen the mice were killed at day 4 post infection. The organs were harvested and homogenized in 800 μl PBS using the FastPrep Homogenizer (MP Biomedicals). After centrifugation at 5,000 x g for 10 min at 4°C the supernatants were analyzed by plaque assay on Vero cells.

### Bioinformatical methods

Nucleoprotein amino acid sequences of HI-Kamigano-25 (MT628438), AfricAII (MT628408), PoTi503 (MT628450), SiAr126 (MT628444), JOSV (HM627173), Oz virus (LC320127), BRBV (MT628414), India (MT628432), BTKV (MT628420), PoTi461 (MT628426) and influenza A/California/07/2009 (H1N1) (NC_026436) were aligned using the aligner MAFFT. The alignment was visualized with Geneious 10.

The 3D structures of the SiAr126 NP monomer and a vRNP-like 24mer were predicted using SWISS-MODEL [82] on the basis of the 3D crystal structures of A/HK/483/97 (H5N1) NP (PDB code: 2q06.2.A (monomer), 4bbl.1.A (oligomer)). The structures of IAV NP and the predicted SiAr126 NP were visualized and colorized using PyMOL.

### Statistical analyses

Data were visualized and statistically evaluated with GraphPad Prism 7. Viral titers of mouse organs were displayed on a log-scale (scatter plot, geometric mean) and viral titers of the growth kinetics displayed as log-transformed values on a linear scale (mean with standard deviation). The polymerase reconstitution assay data were visualized as mean ± standard deviation (n = 3). Weight loss of the animals is shown as mean ± standard error of means. Statistics were computed by a one-way or two-way (for viral growth kinetics) ANOVA using the Tukey’s multiple comparison test. Statistical analyses of viral titers were performed on log-transformed values. The underlying numerical data are deposited in S1 Data.

## Supporting information

S1 Data Underlying numerical data. Excel spreadsheet containing, in separate sheets, the underlying numerical data for figure panels 1A, 1B, 1D, 1E, 1F, 2A, 2B, 2D, 2E, 3B, 3C, 3D, 4A, 4B, 5A, 5B, 5C, 5D, 5E, 7A, 7B, 7C, S1, S2, S5A, S5B.
(XLSX)

S1 Fig Mutations in JOSV NP lead to erratic polymerase activities in the JOSV system. The amino acid substitutions marked in Fig 3A were introduced in JOSV NP and tested in the JOSV polymerase reconstitution system in the absence of MxA. 293T cells were co-transfected with 10 ng of PB1, PB2, PA, 50 ng of the individual NP mutants, 50 ng of pPol-I FF-Luc and 10 ng of RLuc. 24 h after transfection the cells were lysed and firefly and Renilla luciferase activities determined. Firefly luciferase was normalized to Renilla luciferase activity. The relative activity of wildtype NP was set to 100% (mean ± SD, n = 3). Significance was calculated with a one-way ANOVA (Tukey’s multiple comparison test, *p<0,05, ***p<0.001, ns–not significant). wt–wildtype.
(TIFF)

S2 Fig Activity of the individual SiAr126 NP mutants in the presence of MxA or MxA(T103A). 293T cells were co-transfected with 10 ng of PB1, PB2, PA, 50 ng of the individual NP mutants, 50 ng of pPol-I FF-Luc, 10 ng of RLuc and 50 ng of MxA or MxA(T103A) as described in Fig 3C. 24 h after transfection the cells were lysed and the firefly and Renilla luciferase activity determined. Firefly luciferase activity was normalized to Renilla luciferase activity and the expression of NP, actin and MxA was controlled by Western blot. The empty vector control for wildtype SiAr126 NP was set to 100% (mean ± SD, n = 3). Significance was calculated with a one-way ANOVA (Tukey’s multiple comparison test, ***p<0.001, ns–not significant). Wt–wildtype.
(TIFF)

S3 Fig Amino acid alignment of the NPs of Thogotoviruses. NP amino acid sequences of HI-Kamigano-25 (MT628438), AfricAII (MT628408), PoTi503 (MT628450), SiAr126 (MT628444), JOSV (HM627173), Oz virus (LC320127), BRBV (MT628414), India (MT628432), BTKV (MT628420), PoTi461 (MT628426) and influenza A/California/07/2009 (H1N1) (NC_026436) were aligned using the aligner MAFFT. Residues of the NLS in THOV-NP [83] were marked in blue, residues corresponding to positions SiAr126 G327 and R328 were marked in yellow, weak, and red, strong effect on MxA sensitivity, respectively.
(TIFF)

S4 Fig Structure of the SiAr126 NP monomer and oligomer highlighting the positions 327R and 328V. The 3D structures of the SiAr126 NP monomer (A) and a vRNP-like 24mer (B) were predicted (SWISS-MODEL) and rendered using PyMOL. IAV H5N1 NP served as a template (PDB: monomer, 2Q06.2.A; oligomer, 4BBL.1.A).
(TIFF)

S5 Fig Effect of different residues at SiAr126 NP position 328 on MxA sensitivity. (A) Activity of the different NP mutants in the absence of MxA. 293T cells were co-transfected with 10 ng of PB1, PB2, PA, 50 ng of the individual NP mutants, 50 ng of pPol-I FF-Luc and 10 ng of RLuc. At 24 h after transfection the cells were lysed and firefly and Renilla luciferase activities determined. Firefly luciferase activity was normalized to Renilla luciferase activity. The relative activity of wildtype NP R328 was set to 100% (mean ± SD, n = 3). (B) Furthermore, 50 ng of MxA or MxA(T103A) were co-transfected. The empty vector control of the respective NP mutants was set to 100%. The expression of NP, actin and MxA was monitored by Western blot. Significance was calculated by a one-way ANOVA (Tukey’s multiple comparison test, ***p<0.001, ns–not significant). wt–wildtype.
(TIFF)
