# Supplementary figures and images for: Tick-transmitted thogotovirus gains high virulence by a single MxA escape mutation in the viral nucleoprotein
Source: PLoS Pathog. 2020 Nov 16;16(11):e1009038. doi: 10.1371/journal.ppat.1009038 (PMC7704052; doi:10.1371/journal.ppat.1009038)

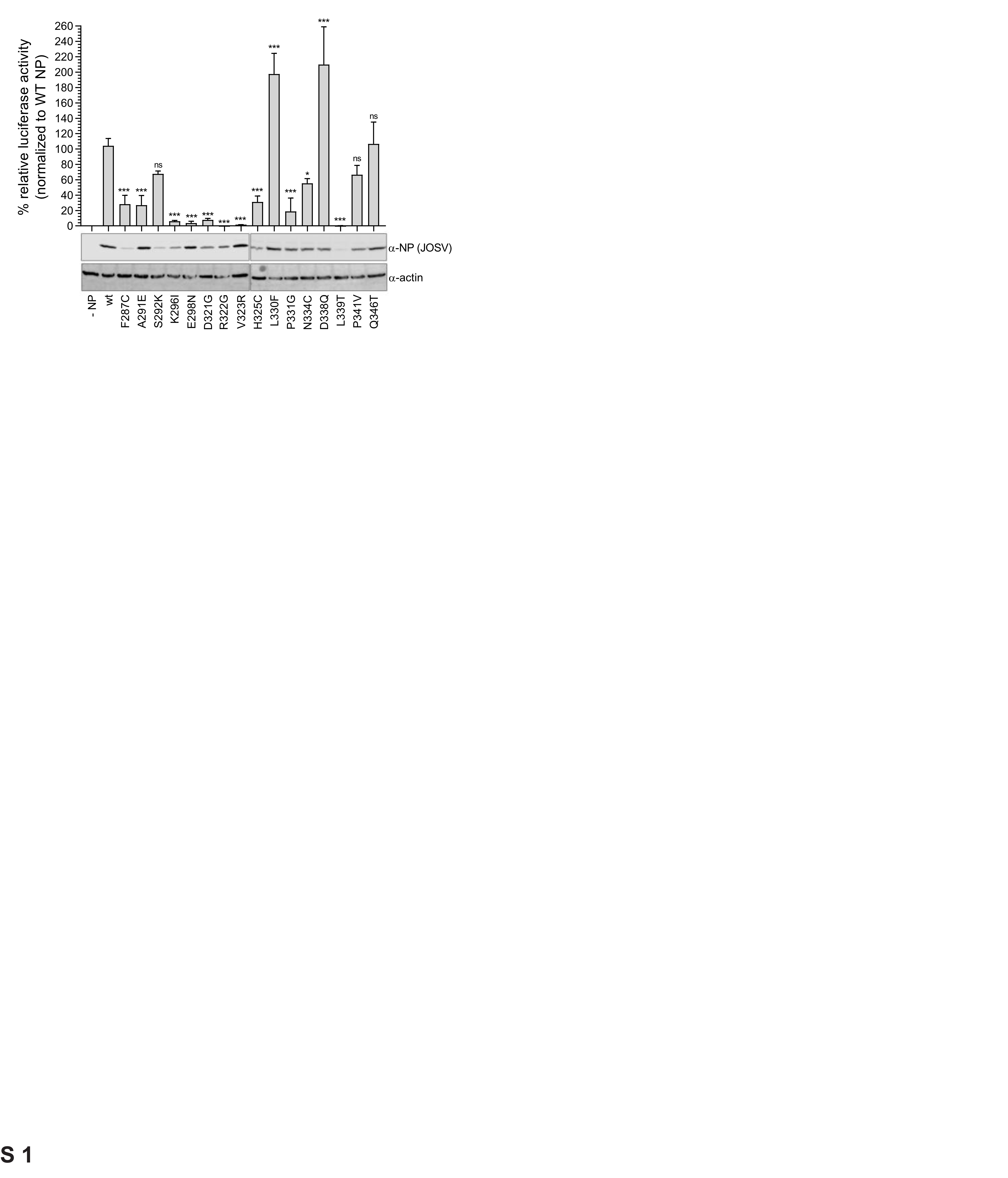

Supplement: S1 Fig — The amino acid substitutions marked in Fig 3A were introduced in JOSV NP and tested in the JOSV polymerase reconstitution system in the absence of MxA. 293T cells were co-transfected with 10 ng of PB1, PB2, PA, 50 ng of the individual NP mutants, 50 ng of pPol-I FF-Luc and 10 ng of RLuc. 24 h after transfection the cells were lysed and firefly and Renilla luciferase activities determined. Firefly luciferase was normalized to Renilla luciferase activity. The relative activity of wildtype NP was set to 100% (mean ± SD, n = 3). Significance was calculated with a one-way ANOVA (Tukey’s multiple comparison test, *p<0,05, ***p<0.001, ns–not significant). wt–wildtype. (TIFF) [file ppat.1009038.s002.tiff]

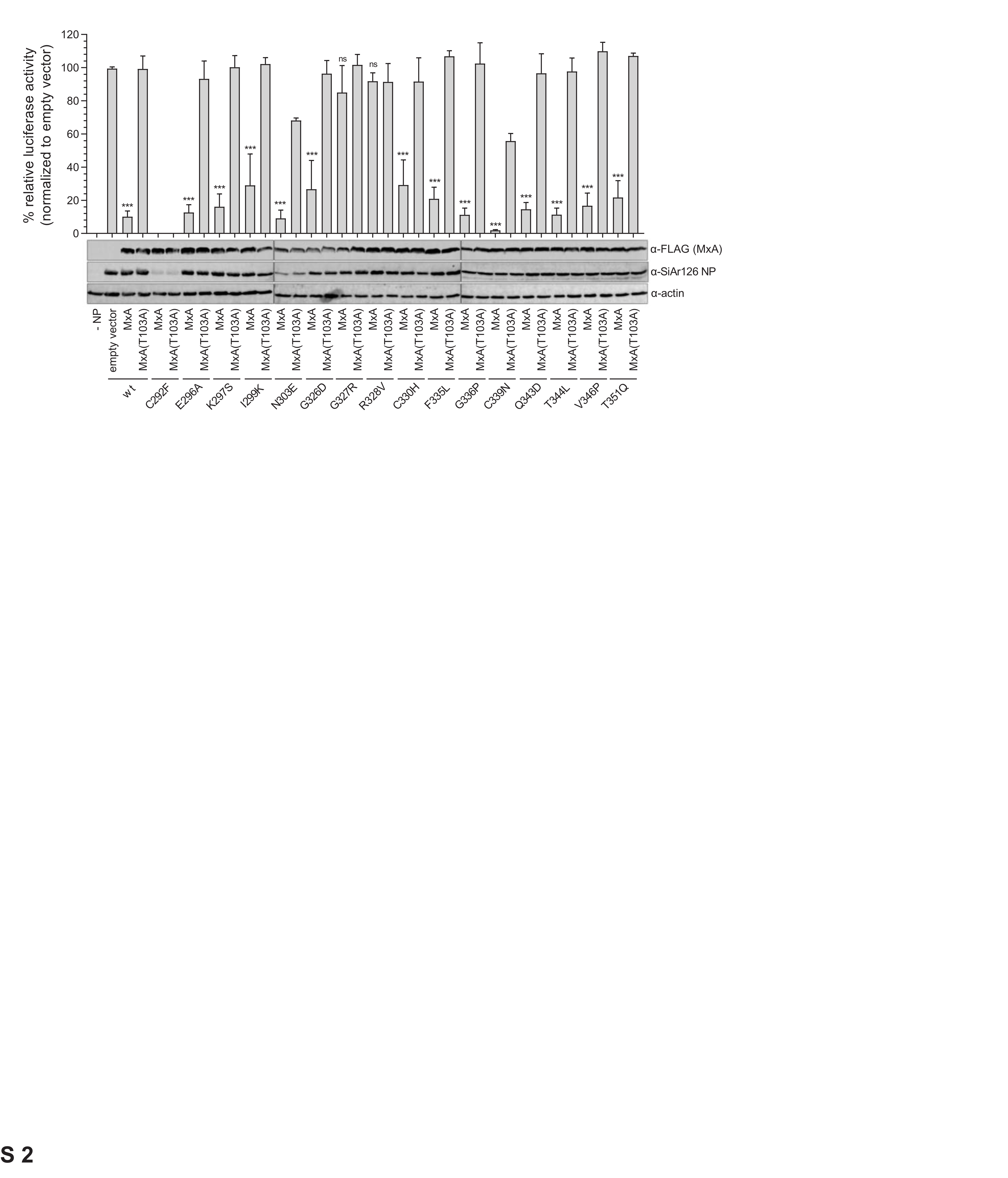

Supplement: S2 Fig — 293T cells were co-transfected with 10 ng of PB1, PB2, PA, 50 ng of the individual NP mutants, 50 ng of pPol-I FF-Luc, 10 ng of RLuc and 50 ng of MxA or MxA(T103A) as described in Fig 3C. 24 h after transfection the cells were lysed and the firefly and Renilla luciferase activity determined. Firefly luciferase activity was normalized to Renilla luciferase activity and the expression of NP, actin and MxA was controlled by Western blot. The empty vector control for wildtype SiAr126 NP was set to 100% (mean ± SD, n = 3). Significance was calculated with a one-way ANOVA (Tukey’s multiple comparison test, ***p<0.001, ns–not significant). Wt–wildtype. (TIFF) [file ppat.1009038.s003.tiff]

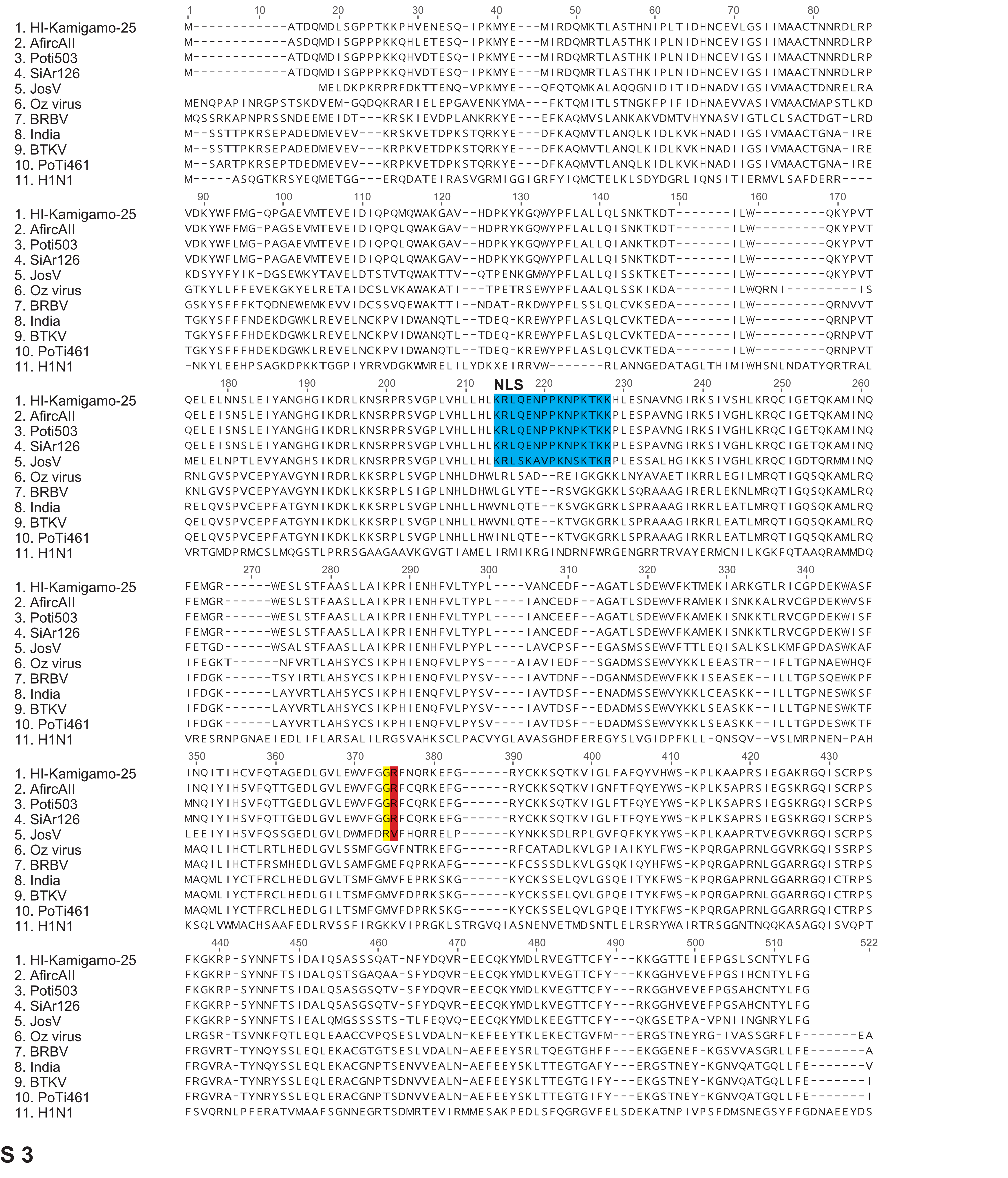

Supplement: S3 Fig — NP amino acid sequences of HI-Kamigano-25 (MT628438), AfricAII (MT628408), PoTi503 (MT628450), SiAr126 (MT628444), JOSV (HM627173), Oz virus (LC320127), BRBV (MT628414), India (MT628432), BTKV (MT628420), PoTi461 (MT628426) and influenza A/California/07/2009 (H1N1) (NC_026436) were aligned using the aligner MAFFT. Residues of the NLS in THOV-NP [83] were marked in blue, residues corresponding to positions SiAr126 G327 and R328 were marked in yellow, weak, and red, strong effect on MxA sensitivity, respectively. (TIFF) [file ppat.1009038.s004.tiff]

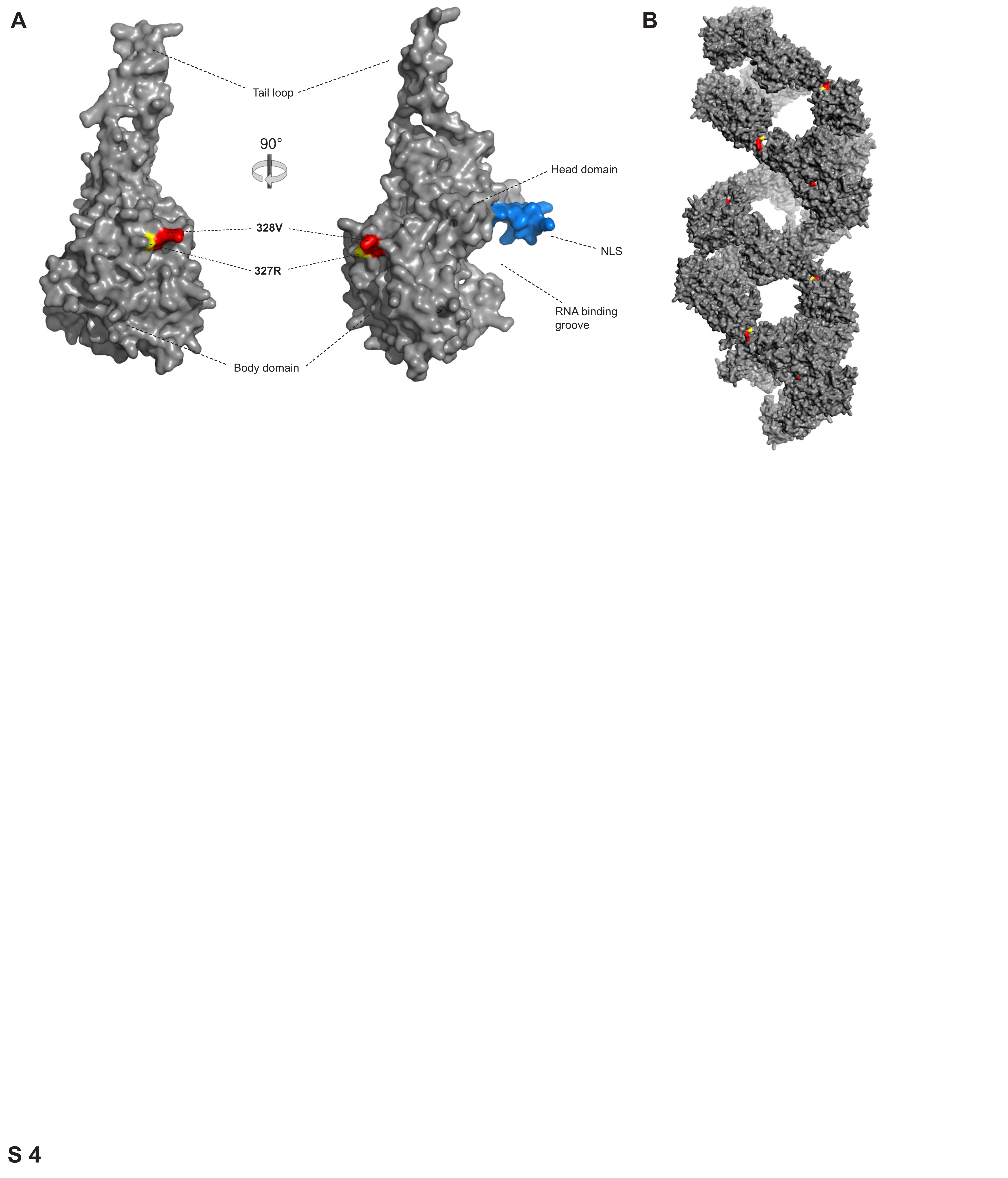

Supplement: S4 Fig — The 3D structures of the SiAr126 NP monomer (A) and a vRNP-like 24mer (B) were predicted (SWISS-MODEL) and rendered using PyMOL. IAV H5N1 NP served as a template (PDB: monomer, 2Q06.2.A; oligomer, 4BBL.1.A). (TIFF) [file ppat.1009038.s005.tiff]

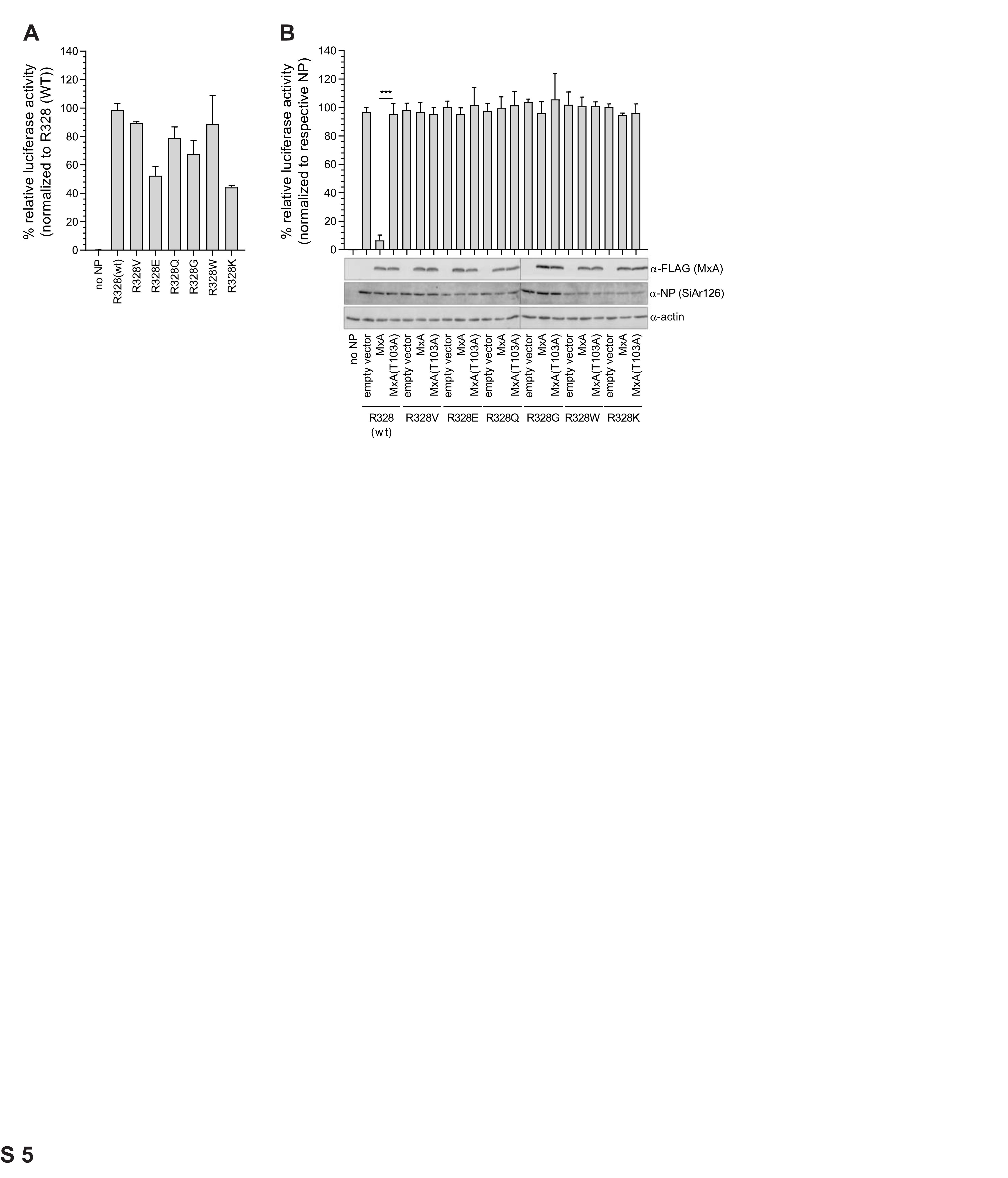

Supplement: S5 Fig — (A) Activity of the different NP mutants in the absence of MxA. 293T cells were co-transfected with 10 ng of PB1, PB2, PA, 50 ng of the individual NP mutants, 50 ng of pPol-I FF-Luc and 10 ng of RLuc. At 24 h after transfection the cells were lysed and firefly and Renilla luciferase activities determined. Firefly luciferase activity was normalized to Renilla luciferase activity. The relative activity of wildtype NP R328 was set to 100% (mean ± SD, n = 3). (B) Furthermore, 50 ng of MxA or MxA(T103A) were co-transfected. The empty vector control of the respective NP mutants was set to 100%. The expression of NP, actin and MxA was monitored by Western blot. Significance was calculated by a one-way ANOVA (Tukey’s multiple comparison test, ***p<0.001, ns–not significant). wt–wildtype. (TIFF) [file ppat.1009038.s006.tiff]
